# Open-Source, Adaptable, All-in-One Smartphone-Based System for Quantitative Analysis of Point-of-Care Diagnostics

**DOI:** 10.3390/diagnostics12030589

**Published:** 2022-02-25

**Authors:** Weronika Schary, Filip Paskali, Simone Rentschler, Christoph Ruppert, Gabriel E. Wagner, Ivo Steinmetz, Hans-Peter Deigner, Matthias Kohl

**Affiliations:** 1 Medical and Life Sciences Faculty, Furtwangen University, 78054 Villingen-Schwenningen, Germany; s.rentschler@hs-furtwangen.de (S.R.); christoph.ruppert@gmail.com (C.R.); hans-peter.deigner@hs-furtwangen.de (H.-P.D.); 2 Institute of Precision Medicine, Furtwangen University, 78054 Villingen-Schwenningen, Germany; 3 Department of Pharmaceutical Chemistry, Pharmaceutical Institute, University of Tuebingen, 72076 Tuebingen, Germany; 4 Institute of Hygiene, Microbiology and Environmental Medicine, Medical University of Graz, 8010 Graz, Austria; gabriel.wagner-lichtenegger@medunigraz.at (G.E.W.); ivo.steinmetz@medunigraz.at (I.S.); 5 EXIM Department, Fraunhofer Institute IZI Leipzig, 18057 Rostock, Germany; 6 Faculty of Science, University of Tuebingen, 72076 Tuebingen, Germany

**Keywords:** point-of-care diagnostics, lateral flow assays, R Shiny application, quantitative image analysis, smartphone-based system

## Abstract

Point-of-care (POC) diagnostics, in particular lateral flow assays (LFA), represent a great opportunity for rapid, precise, low-cost and accessible diagnosis of disease. Especially with the ongoing coronavirus disease 2019 (COVID-19) pandemic, rapid point-of-care tests are becoming everyday tools for identification and prevention. Using smartphones as biosensors can enhance POC devices as portable, low-cost platforms for healthcare and medicine, food and environmental monitoring, improving diagnosis and documentation in remote, low-resource locations. We present an open-source, all-in-one smartphone-based system for quantitative analysis of LFAs. It consists of a 3D-printed photo box, a smartphone for image acquisition, and an R Shiny software package with modular, customizable analysis workflow for image editing, analysis, data extraction, calibration and quantification of the assays. This system is less expensive than commonly used hardware and software, so it could prove very beneficial for diagnostic testing in the context of pandemics, as well as in low-resource countries.

## 1. Introduction

Detection of pathogens or specific biomarkers to identify diseases, ensuring suitable patient care and appropriate therapeutic approaches as well as the assessment and reassessment of therapy according to the patient’s response with the goal of quick recovery—all this depends on precise and rapid diagnostic technologies [1,2,3].

Unfortunately, diagnosis often still involves tedious and expensive testing, requiring costly instruments and trained healthcare or laboratory personnel [3]. With infectious disease epidemics and pandemics, such as the ongoing coronavirus disease 2019 (COVID-19) pandemic, some clinics and laboratories might not possess the required instruments, may be understaffed and face difficulties regarding testing and diagnosing of this and many other diseases [4]. Especially in low-resource countries, the availability of diagnostic tests, laboratories and equipment is often limited. Therefore, the development of rapid, cost-effective diagnostic technologies, which can be performed without fully equipped laboratory facilities, e.g., point-of-care (POC) testing approaches, becomes more and more necessary and requisite [3,5,6,7,8,9,10].

POC testing approaches are performed at the site of patient care, e.g., at the patient’s bedside, and produce results in a quick and straightforward manner without additional processing [3,7,8,11,12]. Lateral Flow Assays (LFAs), a kind of POC testing device containing a carrier material with dry reagents activated by a liquid sample, offer a simple test setup and rapid results in many application areas. These include Cardiology and Haematology, Immunology, Toxicology, Infectious Diseases, Antibiotic Resistance and many more [7,8,11,12]. In the context of health management considering pandemics and epidemics, especially the COVID-19 pandemic, POC testing devices enable quick detection, timely diagnosis and help prevent transmission of infectious diseases [3,9,10,13].

One technology which is already enhancing the field of POC diagnostics is smartphone-based analysis [6,9,10,14]. Smartphones have become almost indispensable for everyday life even in the most remote locations of the world, which makes them an attractive option in diagnostics and research [6,14]. In recent years, smartphone technology has been applied in POC research, utilizing LED flash as an illumination source, built-in cameras as signal detectors and processing capacities [10,14,15].

Using smartphones as biosensors in many different applications (e.g., optical-based methods such as absorbance, reflectance, fluorescence and many more) have potential as portable, low-cost POC platforms for healthcare and medicine, food and environmental monitoring, particularly improving diagnosis and treatment in remote, low-income locations [10,14,16].

We propose a smartphone-based system for the quantification of various lateral flow assays for the detection and diagnosis of diseases. The presented smartphone-based system consists of a 3D-printed photo box for standardized positioning and lighting, a smartphone for image acquisition and an R Shiny [17,18] software package with a modular, customizable analysis workflow for image editing, analysis, data extraction, calibration and quantification. This system is less expensive than commonly used hardware and software for analysis, so it could prove very beneficial for diagnostic testing in the context of pandemics, as well as in low-resource countries, in which laboratory equipment as well as diagnostic facilities are scarce.

The proposed system is facilitated with R Shiny [17,18], an open-source package—free to use and modify. It can be used without extensive programming skills or image analysis software that require longer training periods for the user, which could further the development of diagnostic tools becoming simpler, quicker, more efficient, and hence even more cost-effective compared to the gold standard methods used in detection and diagnosis today. Also, the automatic documentation of all analysis steps, implemented in the application via R Markdown [19], allows for accurate reproducibility which is of high importance in biomedical research and clinical practice [20,21]. In comparison with other smartphone-based systems for POC applications [16,22,23,24] our system is not limited to the detection of one type of band-based POC device only but can be used with any band-based POC device.

## 2. Materials and Methods

### 2.1. Image Acquisition

The first component of our proposed system is a 3D-printed photo box, as proposed by Mahmoud et al. 2020, which consists of a 3D-printed construction with a 365 nm UV-LED light source consisting of a UV-LED, Ø 50 mm aluminium heatsink, 350 mA power supply, and power supply cable with a switch for connection to the European standard 220 V power grid [25]. The UV-LED light source was used for the excitation of quantum dot-labelled lateral flow assays from Ruppert et al. 2020 [26] and Mahmoud et al. 2021 [25].

The photo box was designed with an adapter for use with a Huawei P30 Pro (Huawei Device Co., Ltd., Shenzhen, China) smartphone, however, a modification of the adapter to use a different smartphone model could be done easily. The CAD files for the 3D printing of the photo box can be downloaded from GitHub (https://github.com/fpaskali/LFApp/tree/main/Photobox (accessed on 21 February 2022)). Images of the used test strips were acquired via smartphone camera (iPhone 5S (Apple Inc., Cupertino, California, USA), Huawei P30 Pro) and BioImager “ChemStudioPLUS” (Analytik Jena GmbH, Jena, Germany), but basically any other device can be used for image acquisition.

### 2.2. R Package Development and Functionality of the Apps

For further image analysis, the package *LFApp* was developed to enable image editing, cropping, segmentation, background correction, data analysis, calibration and quantification. The development of the applications was carried out using Shiny [18] and R [17]. Besides Shiny, other major packages used are EBImage [27], a package for image manipulation, ggplot2 [28], used for data visualization, DT [29] for generating HTML tables with built-in usability features. Other notable packages used are: shinyjs [30], stats [17], mgcv [31], shinyFiles [32], fs [33], rmarkdown [19] and shinythemes [34]. Furthermore, we designed an additional version of the UI module, using shinyMobile [35], to make the app more accessible on a small touchscreen.

To perform background correction and quantification of the signal in color images, there is a need to transform the image to grayscale mode. This transformation is carried out by the EBImage channel function and particular mode. There are two options to convert an RGB colour image to grayscale, namely the luminance preserving colour approach (0.2126 × R + 0.7152 × G + 0.0722 × B) and the gray approach (1/3 × R + 1/3 × G + 1/3 × B). Finally, one can choose one of the RGB channels to filter out unwanted colour channels and extract the pixel values of that channel which will then be used for quantification [27]. Additionally, inversion was added to solve the problem of band signals with lower intensities than background. The inversion mode generates the negative of the image, in which each pixel of the original image is subtracted from 1.

For background correction, the four following methods are included in the application. The Otsu method finds the ideal threshold level that separates the pixels in two classes and works best when the histogram of the image is bimodal [36,37]. The Li method is an iterative algorithm that calculates the threshold that minimizes the cross entropy of original and segmented image [38,39]. Quantile is a semi-automatic method, performed by computing an empirical quantile from the intensity values of the segments without bands. The value of the respective empirical quantile is used as a background threshold. Another semi-automatic method is Triangle [40], which is a geometric global search algorithm that works best when the histogram of the image is unimodal and skewed. For precise threshold selection, an offset parameter can be added to the calculated threshold.

The fitting of the calibration curve is performed by applying function “lm” or “loess” of package stats [17], or “gam” of package mgcv [31], enabling the use of linear, local polynomial or general additive models for calibration analysis. The respective model can be specified in side panel of the calibration tab. The calibration curve is plotted by the package ggplot2 [28]. Additionally, R^2^ (only in case of lm), limit of blank (LOB), limit of detection (LOD) and (lower) limit of quantification ((L)LOQ) are computed from the calibration curve and printed [41,42].

### 2.3. Analyzed Assays

During the development and optimization of the application and our photo box system, a number of LFAs were used, namely Melioidosis DS rapid test by Wagner et al. 2020 [43], gold nanoparticle LFA for digoxigenin monitoring by Ruppert et al. 2019 [44], duplex quantum dot labelled LFA for C-reactive protein and interleukin-6 quantification by Ruppert et al. 2020 [26] and duplex quantum dot labelled LFA for thrombin and interleukin-6 quantification by Mahmoud et al. 2020 [25]. The image acquisition and segmentation module are very flexible, and it can analyse band-like structures in any kind of assay images.

## 3. Results

Our goal was to build a versatile, universally applicable free open-source system, that is scalable and extensible, and also modifiable to suit a wide range of research and diagnostic purposes. To address these needs, Shiny [18], an R [17] package for building interactive web applications, was chosen. The advantages of using Shiny are portability and easy deployment of the applications. In combination with a 3D-printed photo box [25], it represents an all-in-one, portable, cost-efficient and easily reproducible setup for extensive analysis, that works well on computers as well as portable devices, such as smartphones. The system was optimized and validated with images of several different types of lateral flow assays, such as a Dipstick assay by Wagner et al. [43], a Digoxigenin lateral flow assay (LFA) and a duplex quantum dot labelled LFA by Ruppert et al. [26,44], as well as a multicolour quantum dot labelled LFA by Mahmoud et al. [25]. Shiny mobile version [35] is directly useable on smartphones, allowing a quick and detailed analysis of LFAs in any setting. Our LFA R Shiny application performed equally well to or outperformed the methods proposed in other studies. The results of the comparison of the analyses are shown in Table 1, Supplementary Materials Table S1 and Figure S1.There is one exception in the calibration of thrombin where Mahmoud et al. used a non-linear model, whereas we fitted a simple linear model.

Our software package (available at: https://github.com/fpaskali/LFApp and https://cran.r-project.org/package=LFApp (accessed on 21 February 2022)) consists of four modular R Shiny applications:

(1) *LFA App core* for image acquisition, editing, region of interest definition via gridding, background correction with multiple available methods, as well as intensity data extraction of the pre-defined bands of the analysed LFAs;
(2) *LFA App calibration*, which also includes tools for data processing, adding additional information and calibration functionality;
(3) *LFA App quantification*, which enables quantification of the extracted intensity values via loading existing calibration models;
(4) *LFA App analysis*, which includes all the modules mentioned above and enables full analysis in one application.

**Figure 1:**
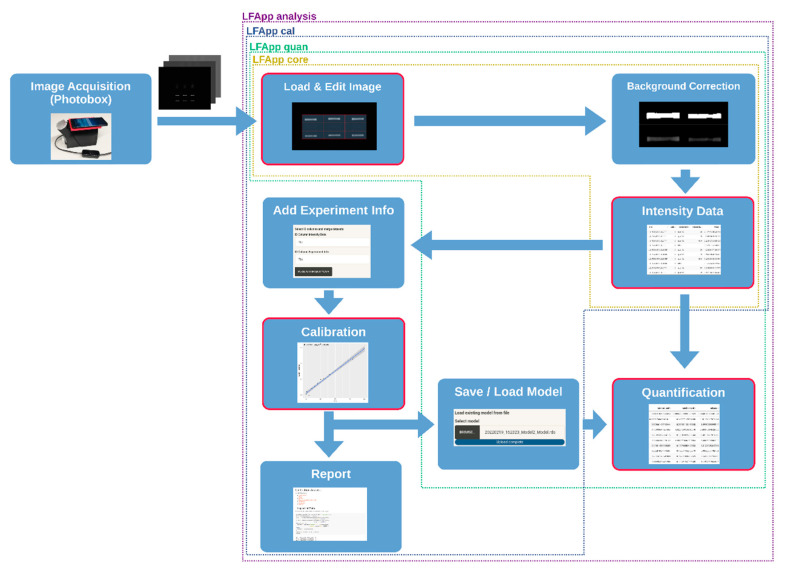
An overview of the content and workflow of our system. The different coloured frames indicate which modules are included in which application. The red border of modules represents optional starting points in the workflow.

The graphical user interface of the apps is built in a modular setup divided in several tabs, where each tab represents a specific step of the workflow (see Figure 1). While the applications can be used in a sequential fashion, the specific steps can be carried out individually.

**Figure 2:**
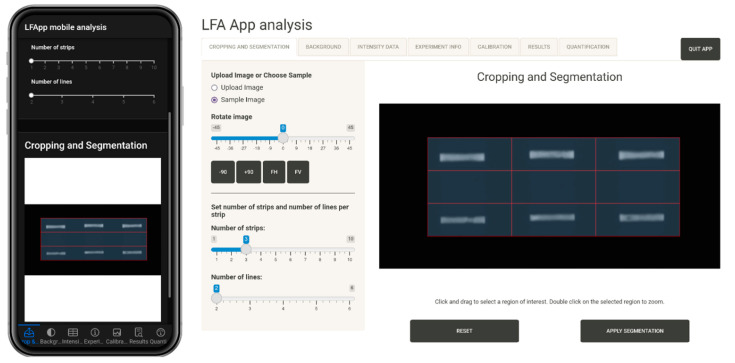
Image acquisition and editing functionality in the cropping and segmentation Tab of the LFA App mobile analysis (**left**) and LFA App analysis (**right**).

The first tab consists of the image loading and region of interest grid definition. For image loading and manipulation, we utilized the R package EBImage [27], supporting standard image types. Here, the image can be rotated or flipped horizontally and vertically and cropped to a specific size. The type of the grid can be specified in the side panel, according to the type of the assay, by selecting the number of lines (signal bands on the LFA) and strips (number of LFAs in the image). Then, the region of interest can be precisely marked on the interactive plot. Figure 2 shows an example with the sample image of the application, in which a grid for two lines and three strips is used. The result is a 3 × 3 grid, because an extra rectangle is generated between the two lines of each strip that may later be used for background correction (quantile method). Image manipulation is carried out in a non-destructive fashion, allowing one to restore the original state at any time during analysis. When editing is completed to the user’s satisfaction and the grid is defined, segmentation is enabled and the application proceeds to the next tab.

The second tab is background correction of the gridded image, done after the selection of the region of interest. This module allows us to enhance the signal and to clear the image by reducing noise and false positive pixels. For this purpose, various algorithms are used in the application. Otsu [36,37] and Li [38,39] are non-parametric, fully automatic algorithms that find the optimal threshold for the image. Additionally, two semi-automatic algorithms, namely Quantile and Triangle [40], were included, to cover different images and cases where automatic threshold results are not ideal. The automatic thresholding methods do not require additional parameters, while the semi-automatic methods have an additional parameter that can be tuned for more flexible thresholding. These settings will be saved in a data table for documentation and to make the analysis reproducible.

**Figure 3:**
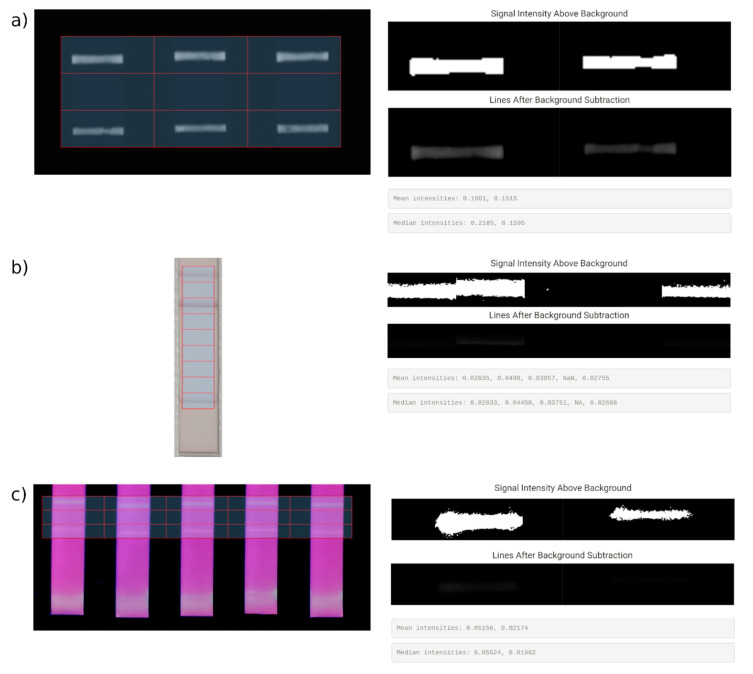
Background correction with three example images; (**a**) a grayscale LFA image acquired via BioImager (**left**) and its background correction results (**right**), (**b**) a colour LFA image taken with a smartphone (**left**) and its background correction results (**right**) and (**c**) a multicolour LFA image acquired with our smartphone imager (**left**) and its background correction results (**right**).

The background correction process begins with the selection of the strip that is going to be analysed. The maximal number of strips is defined in the first tab as described above. The implemented thresholding methods work with grayscale images; hence, two grayscale conversion methods are implemented, namely a luminance and a gray approach. Colour images can also be analysed by selecting one of the three colour channels (RGB). The application also provides colour inversion functionality, which proves beneficial in the analysis of LFAs with fluorescent labelling, in which the lines or bands can be lighter than the background. After the thresholding method is applied, several info and plot boxes are rendered in the main panel of the second tab. Regarding Figure 3, first the individual threshold levels are displayed. The upper plots show the pixels above the threshold from the lines of the selected strip, whereas the lower plots display the signal after background subtraction. Below, the calculated mean and median intensities of the lines in order from the top of the strip to the bottom are shown. The values can be added to an intensity data table and the user is able to continue with the background correction and quantification of other strips or images.

In the third tab, using the DT package [29] a table of intensity data is rendered, that can be easily filtered and sorted. It contains all data from the quantification, such as file name, the number of strips, background correction method, additionally applied parameters, as well as the mean and median values of all lines of the analysed strip. The measured intensity data can be saved or deleted to restart. Existing intensity data can be imported and the current data can be added. *LFA App core* consists of these three tabs representing the core functionality of our software package—image analysis and intensity data extraction.

*LFA App calibration* contains data processing functionality in the *Experiment Info* tab, where information about the experiment can be merged with measurements performed with the application. It is also an optional starting point in case of analysis of pre-existing data. Also, an already merged data set can be loaded using the upload widget in the sidebar and data sets can be adjusted with tools from the sidebar, when experiment data is stored in a non-standard .csv file. After the upload of experiment data, it can be merged with intensity data by specifying the name of the columns of intensity data and experiment data accordingly. As in the previous tab, the data can be saved or deleted for restart. In the main panel of the *Experiment Info* tab, the data table with experiment data or a combined table is shown. Additionally, *LFA App calibration* has the *Calibration* tab, which bundles different functionalities, such as averaging technical replicates and tools for reshaping the table from long to wide, to pre-process the data before calibration. The calibration can be performed by using combined data from the previous tab, or by loading previously saved data. Additionally, the calibration can be performed on a subset of the data, by inputting a logical R expression in the *specify subset* field. Finally, the calibration module uses the power of R to compute a variety of calibration models fitted with functions “lm” (linear models) and “loess” (local polynomial regression) of R package stats [17] as well as function “gam” (generalized additive models) of R package mgcv [31].

**Figure 4:**
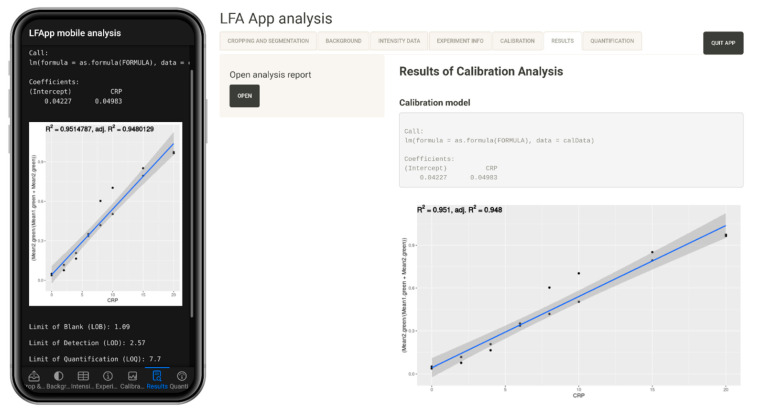
Results of the calibration analysis with LFA App mobile analysis (**left**) and LFA App analysis (**right**).

The results of the calibration are displayed in the sixth tab (see Figure 4), with specific calibration values, such as R^2^ (only in case of lm), limit of blank, limit of detection and limit of quantitation [41,42].

Furthermore, at the end of the calibration analysis, a full report is generated by R Markdown [19], summarizing the fitting of the calibration model. Full reports generated for the lateral flow assays of Ruppert et al. 2019 [44], Ruppert et al. 2020 [26] and Mahmoud et al. 2020 [25] can be found in the supplement; including side-by-side comparison of LOB, LOD, LOQ values as well as calibration curves for each analysis.

*LFA App quantification* offers quantification of substrates via calibration models and extracted intensity data values. In the *Quantification* tab, a calibration model can be specified to quantify the intensity data values as concentrations. Also, a data table (similar to the *Intensity Data* tab) is displayed with the addition of the calculated concentration values for each measurement of the image analysis as well as the calibration model.

*LFA App analysis* offers the functionality of all modules combined into one.

The modularity of the software package allows for flexible approaches in the image and data analysis as well as different starting points. The user can start with the image analysis in the application or use extracted intensity values from different software and continue with data analysis steps. Calibration can be performed for a sample assay and the calibration model can be exported and re-applied for the quantification of many different assays in an experimental setup. Due to the flexible implementation, the different modules can easily be adjusted to the researcher’s needs or recombined to create new applications.

## 4. Discussion

Smartphones accompany us through our everyday lives. The potential of smartphones as inexpensive research instruments or in telemedicine or telehealth is currently gaining pace.

Our proposed smartphone-based system for the quantification of LFAs and other POC devices represents another step in the way of using smartphones as biosensors in the field of medicine, healthcare or food and environmental monitoring. The 3D-printed photo box used for this system has been developed out of a black box with an iPhone 5S and used for prior testing in a previous publication [44]. Since then, it has been tested extensively with various LFA tests [25,26,43,44].

This self-contained system can be used directly for image acquisition, editing, analysis and obtaining data from diagnostic LFAs as an all-in-one solution on a smartphone. Furthermore, the application itself can be used on a smartphone as well as on a computer as analysis software for images of LFAs from other hardware, e.g., BioImagers, and with data obtained with different software. As stated in previous publications [25,26,44], the results and analysis obtained with the smartphone-based system are equal or superior to other hardware and analysis software. Our proposed system enables the analysis of many different assays and produces high-quality results under complex conditions, such as low saturation or blurred bands. Our software allows easy and automatic documentation of steps and settings and ensures intuitive handling even for non-tech savvy users. It is created with a straightforward design and is completely adjustable to the requirements of the user or research team. We have shown that our system is compatible with many LFA experiments and enables quick, portable and cost-effective analysis.

Especially in context of the ongoing COVID-19 pandemic, our system combined with a functional LFA or other POC device testing for SARS-CoV-2 could provide a major step forward in the field of telemedicine and at-home diagnostic tools, as a false test-read out is still one of the main issues of point-of-care rapid tests. This holds true especially in low-resource areas, where laboratories, equipment and personnel are lacking. Many different application possibilities for this system include at-home testing with POC devices for other infectious diseases, regular at-home testing for chronic diseases, as well as periodic testing for food and environmental safety. Additionally, various applications in laboratory or research environments, such as documentation and quantification of electrophoresis experiments, can be achieved. There are almost no limitations to our application and our system in terms of adaptation to a specific diagnostic or scientific approach, as well as other types of assays or POC devices.

Smartphones as biosensors can be prone to technical difficulties and artifacts due to different lighting or positioning of the test strips. These problems can be reduced by providing a standardized lighting and fixed position of the test strips inside the 3D-printed photo box and by rotation and cropping functionality of the analysis application. Nevertheless, smartphones as closed systems could provide limitations because of internal differences of camera modules or processors that might cause fluctuating outcomes when it comes to image acquisition and, therefore, associated further analysis.

One approach to overcome this issue could be to set up smartphone cameras with a standardized calibration before image acquisition of the tests, e.g., via the proposed standardized methodology for the calibration of consumer cameras by Burggraaff et al. [45].

For the future, the problem of differences in cameras and processors of smartphones, could be omitted with the use of small form factor computers and standardized hardware to create a complete, low-cost, scanning hardware and software system. Also, regarding the certification of such devices for use in regulated environments, for example in clinics or certified diagnostic laboratories, a low-cost computer system could prove advantageous.

Optimization and further improvements of our system and application are of utmost importance to us and we will continue to innovate this system to make it accessible to as many users as possible for quick, easy and cost-effective image and data analysis in their fields of research or applications. We are currently developing applications for assays in array format such as lateral flow microarrays, but also for protein microarrays and others.

## Figures and Tables

**Table 1:** Side-by-side performance comparison of the results of our system and the methods of other studies using R^2^ goodness of fit.

Study	Response (y-Axis)	R^2^ (Study)	R^2^ (LFApp)
Digoxigenin calibration (Iphone 5S), Ruppert et al., 2019 [44]	cl/tl	0.96	0.97
Digoxigenin serum (Iphone 5S), Ruppert et al., 2019 [44]	cl/tl	0.93	0.97
Digoxigenin calibration (Bioimager),Ruppert et al., 2019 [44]	cl/tl	0.96	0.99
Digoxigenin serum (Bioimager),Ruppert et al., 2019 [44]	cl/tl	0.97	0.99
Thrombin (Huawei P30 Pro), Mahmoud et al., 2020 [25]	tl	0.99 *	0.95
IL-6 (Huawei P30 Pro), Mahmoud et al., 2020 [25]	tl/cl	0.95	0.95
CRP (Bioimager), Ruppert et al., 2020 [26]	tl/(tl + cl)	0.95	0.95
IL-6 (Bioimager) Ruppert et al., 2020 [26]	tl/(tl + cl)	0.97	0.97

cl = control line; tl = test line; * reported R^2^ is calculated by fitting non-linear model.

## Data Availability

The complete R code developed in this project is included in our open-source package LFApp. The developmental version is available on GitHub (https://github.com/fpaskali/LFApp (accessed on 21 February 2022)) under L-GPL-3 license and the stable version is available on CRAN (https://cran.r-project.org/package=LFApp (accessed on 21 February 2022)) the comprehensive R archive network. The desktop and mobile version of our *LFA App analysis* can be tested on shinyapps.io provided by RStudio (https://www.shinyapps.io/ (accessed on 21 February 2022)), where the two versions of our app are at https://lfapp.shinyapps.io/LFAnalysis/ and https://lfapp.shinyapps.io/mobile_app/ (accessed on 21 February 2022), respectively. Package LFApp also includes our user guide in form of a so-called vignette (available also at: https://fpaskali.github.io/LFApp/ (accessed on 21 February 2022)). YouTube videos demonstrating the installation and use of the package are available at https://www.youtube.com/playlist?list=PLRgOZXM8LZ0gJwtsFNxBiu9WJG1TJjFuP (accessed on 21 February 2022). The CAD files for the 3D printing of the photo box can be downloaded from GitHub (https://github.com/fpaskali/LFApp/tree/main/Photobox (accessed on 21 February 2022)).

## Supplementary Materials

The following are available online at https://www.mdpi.com/article/10.3390/diagnostics12030589/s1, Figure S1 and Table S1.
