# Supplementary material for: Open-Source, Adaptable, All-in-One Smartphone-Based System for Quantitative Analysis of Point-of-Care Diagnostics
Source: Diagnostics (Basel). 2022 Feb 25;12(3):589. doi: 10.3390/diagnostics12030589 (PMC8947044; doi:10.3390/diagnostics12030589)
Supplement: Supplementary file 1 [file diagnostics-12-00589-s001.zip › diagnostics-1600132-Supplementary/diagnostics-1600132-Supplementary.pdf]

Study	LOB nM (study)	LOB nM (LFApp)	LOD nM (study)	LOD nM (LFApp)	LOQ nM (study)	LOQ nM (LFApp)
**Ruppert et al., 2019 Digoxigenin calibration (iPhone 5S)**	13,1	8,21	31,3	28	123,7	84
**Ruppert et al., 2019 Digoxigenin serum (iPhone 5S)**	29,2	4,59	39,4	11,6	77,2	34,9
**Ruppert et al., 2019 Digoxigenin calibration (Bioimager)**	17,2	5,01	29,7	17,1	86,8	51,2
**Ruppert et al., 2019 Digoxigenin serum (Bioimager)**	29,2	3,15	39,4	7,97	77,2	23,9
**Mahmoud et al., 2020 Thrombin (Huawei P30 Pro)***	na	3,12	3	12,6	na	37,9
**Mahmoud et al., 2020 IL-6 (Huawei P30 Pro)**	na	9,55	100	38,6	na	116
**Ruppert et al., 2020 CRP (Bioimager)**	0,33	1,09	1,27	2,57	5,52	7,7
**Ruppert et al., 2020 IL-6 (Bioimager)**	0,46	0,792	2,28	1,86	7,33	5,58

Supplementary Table S1. Side-by-side comparison of LOB, LOD, LOQ values computed by our system and the reported values of other studies.
*reported values are calculated by fitting non-linear calibration model.

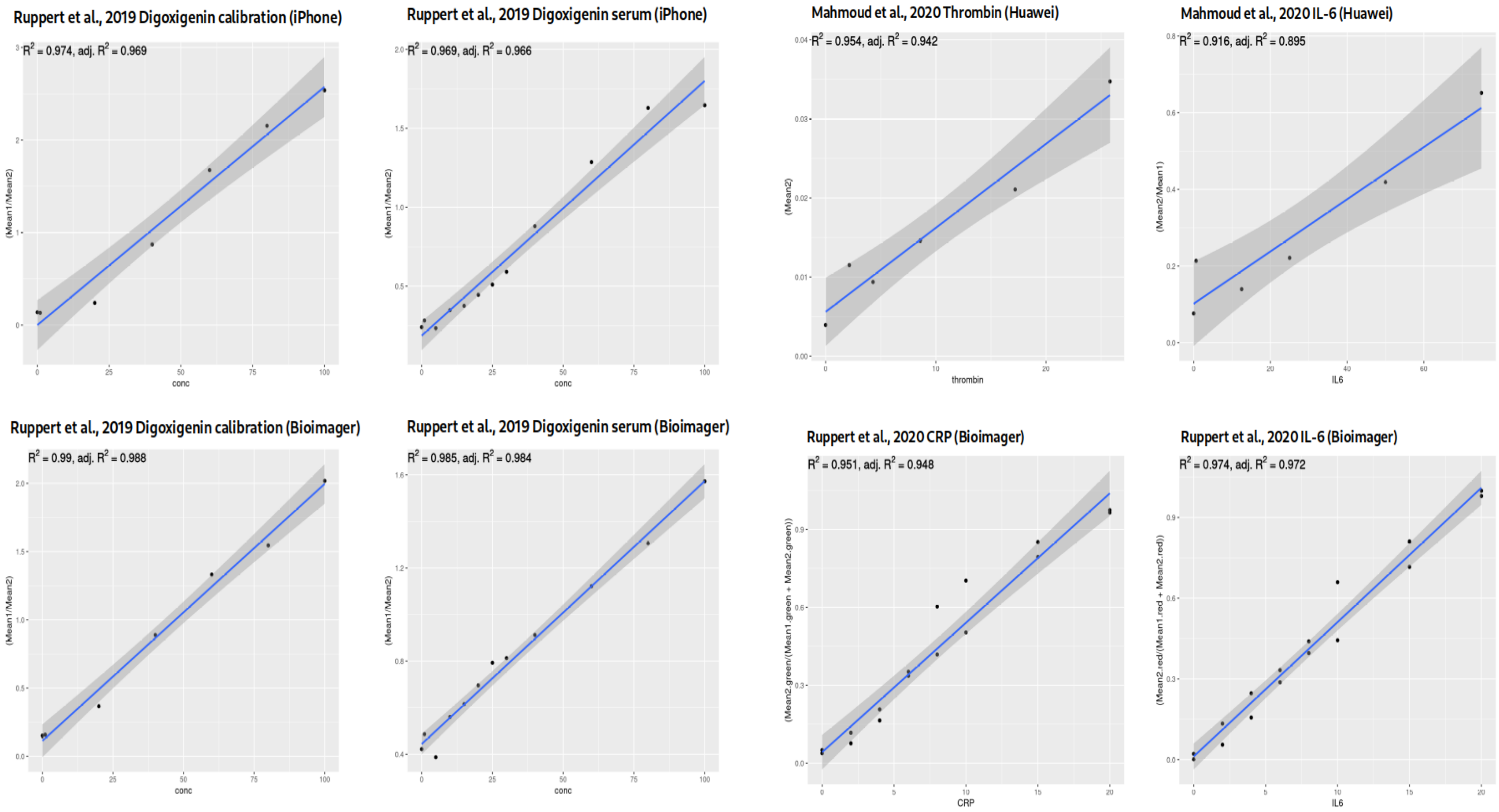

Supplementary Figure S1. Calibration curves computed by LFApp with the data provided by Ruppert et al. 2019, Ruppert et al. 2020 and Mahmoud et al. 2020.
