# Supplementary material for: Open-Source, Adaptable, All-in-One Smartphone-Based System for Quantitative Analysis of Point-of-Care Diagnostics
Source: Diagnostics (Basel). 2022 Feb 25;12(3):589. doi: 10.3390/diagnostics12030589 (PMC8947044; doi:10.3390/diagnostics12030589)
Supplement: Supplementary file 1 [file diagnostics-12-00589-s001.zip › diagnostics-1600132-Supplementary/Reports.pdf]

Calibration Analysis

18 February 2022

Contents

1 Import of Data **1**

2 Model **2**

3 Analysis **2**

4 Computation of LOB, LOD and LOQ **3**

5 Save Results **4**

6 Save Model **4**

7 Software **4**

1 Import of Data

We import the data and select the variables needed for the analysis.

```
load(file.path(PATH.OUT, paste0(FILENAME, "_Data.RData")))
tmp <- strsplit(FORMULA, "~")[[1]]
y.var <- names(unlist(sapply(colnames(CalibrationData),
                             grep, x = tmp[1])))
x.vars <- names(unlist(sapply(colnames(CalibrationData),
                              grep, x = tmp[2])))
if(SUBSET != ""){
  calData <- eval(call("subset", x =  CalibrationData,
                       subset = parse(text = SUBSET)))
}else{
  calData <- CalibrationData
}
calData <- calData[,c(y.var, x.vars)]
calData
```

```
##         Mean1      Mean2 conc
## 7 0.011024184 0.07950319    0
## 1 0.009709756 0.07315447    1
## 3 0.014133376 0.05891563   20
## 4 0.032329452 0.03707950   40
## 5 0.045591884 0.02722176   60
## 6 0.046312130 0.02150471   80
## 2 0.047471047 0.01871509  100
```

2 Model

We will apply the following model.

```
FORMULA
```

```
## [1] "(Mean1 / Mean2) ~ conc"
```

3 Analysis

We now fit (simple) linear model.

```
fit <- lm(as.formula(FORMULA), data = calData)
summary(fit)
```

```
##
## Call:
## lm(formula = as.formula(FORMULA), data = calData)
##
## Residuals:
##        7        1        3        4        5        6        2
##  0.13820  0.10653 -0.27518 -0.15779  0.13054  0.09469 -0.03699
##
## Coefficients:
##              Estimate Std. Error t value Pr(>|t|)
## (Intercept) 0.0004675  0.1048599   0.004    0.997
## conc        0.0257303  0.0018704  13.757 3.64e-05 ***
## ---
## Signif. codes:  0 '***' 0.001 '**' 0.01 '*' 0.05 '.' 0.1 ' ' 1
##
## Residual standard error: 0.178 on 5 degrees of freedom
## Multiple R-squared:  0.9743, Adjusted R-squared:  0.9691
## F-statistic: 189.2 on 1 and 5 DF,  p-value: 3.643e-05
```

We determine the inverse of the fitted model.

```
ab <- coef(fit)
names(ab) <- NULL
predFunc <- function(newdata){}
body(predFunc) <- substitute({ with(newdata, (eval(y)-a)/b) },
                             list(y = parse(text = respVar),
                                a = ab[1],
                                b = ab[2]))
```

We plot the given concentrations against the fitted values.

```
library(ggplot2)
modelPlot <- ggplot(calData, aes_string(x = concVar, y = respVar)) +
            geom_point() + geom_smooth(method = "lm") +
            annotate("text",  x=-Inf, y = Inf,
            label = substitute(paste(R^2, " = ", R2, ", adj. ", R^2, " = ", adj.R2),
                              list(R2 = signif(summary(fit)$r.squared,3),
                                   adj.R2 = signif(summary(fit)$adj.r.squared, 3))),
            vjust=1, hjust=0, size = 5)
modelPlot
```

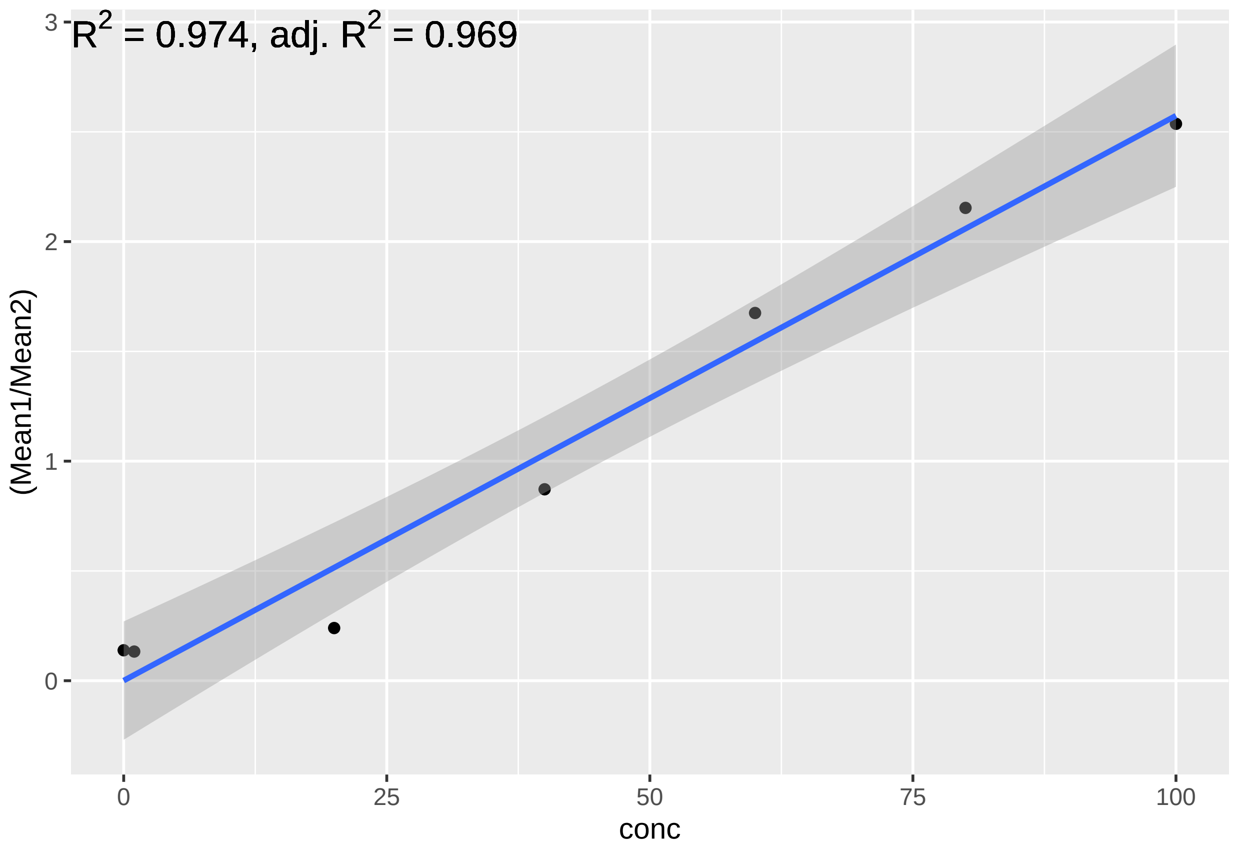

4 Computation of LOB, LOD and LOQ

We compute limit of blank (LOB), limit of detection (LOD) and limit of quantification (LOQ) by inverting the regression fit. We get the LOB by inverting the upper bound of the one-sided 95% confidence interval at concentration 0. In case of LOD, the upper-bound of the 99.95% confidence interval at concentration 0 is inverted. LOQ is determined as $3 \times$ LOD.

```
if(ab[2] > 0){
  LOB <- (confint(fit, parm = 1, level = 0.90)[2]-ab[1])/ab[2]
  LOD <- (confint(fit, parm = 1, level = 0.999)[2]-ab[1])/ab[2]
}else{
  LOB <- (confint(fit, parm = 1, level = 0.90)[1]-ab[1])/ab[2]
  LOD <- (confint(fit, parm = 1, level = 0.999)[1]-ab[1])/ab[2]
}
names(LOB) <- "LOB"
names(LOD) <- "LOD"
LOQ <- 3*LOD
names(LOQ) <- "LOQ"
LOB
```

```
##      LOB
## 8.212011
```

```
LOD
```

```
##      LOD
## 27.99282
```

```r
LOQ
```

```
##      LOQ
## 83.97845
```

5 Save Results

We save the results.

```r
save(fit, LOB, LOD, LOQ, file = file.path(PATH.OUT, paste0(FILENAME, "_Results.RData")))
```

6 Save Model

We save the inverse of the fitted model to be able to apply it for predicting concentrations.

```r
saveRDS(object = predFunc, file = file.path(PATH.OUT, paste0(FILENAME, "_Model.rds")))
```

7 Software

```r
sessionInfo()
```

```
## R version 4.1.2 (2021-11-01)
## Platform: x86_64-pc-linux-gnu (64-bit)
## Running under: Arch Linux
##
## Matrix products: default
## BLAS:   /usr/lib/libopenblasp-r0.3.19.so
## LAPACK: /usr/lib/liblapack.so.3.10.0
##
## locale:
##  [1] LC_CTYPE=en_US.UTF-8       LC_NUMERIC=C
##  [3] LC_TIME=en_US.UTF-8        LC_COLLATE=en_US.UTF-8
##  [5] LC_MONETARY=en_US.UTF-8    LC_MESSAGES=en_US.UTF-8
##  [7] LC_PAPER=en_US.UTF-8       LC_NAME=C
##  [9] LC_ADDRESS=C               LC_TELEPHONE=C
## [11] LC_MEASUREMENT=en_US.UTF-8 LC_IDENTIFICATION=C
##
## attached base packages:
## [1] stats     graphics  grDevices utils     datasets  methods   base
##
## other attached packages:
## [1] ggplot2_3.3.5 shiny_1.7.1
##
## loaded via a namespace (and not attached):
##  [1] Rcpp_1.0.8          locfit_1.5-9.4      lattice_0.20-45
##  [4] fftwtools_0.9-11    png_0.1-7           assertthat_0.2.1
##  [7] digest_0.6.29       utf8_1.2.2          mime_0.12
## [10] R6_2.5.1            tiff_0.1-10         evaluate_0.14
## [13] highr_0.9           pillar_1.7.0        rlang_1.0.1
## [16] fontawesome_0.2.2   jquerylib_0.1.4     Matrix_1.3-4
## [19] DT_0.20             rmarkdown_2.11      labeling_0.4.2
## [22] shinythemes_1.2.0   splines_4.1.2       shinyjs_2.1.0
```

```
## [25] LFApp_1.3           stringr_1.4.0       htmlwidgets_1.5.4
## [28] RCurl_1.98-1.5      munsell_0.5.0       xfun_0.29
## [31] compiler_4.1.2      httpuv_1.6.5        pkgconfig_2.0.3
## [34] BiocGenerics_0.40.0 mgcv_1.8-38         htmltools_0.5.2
## [37] tidyselect_1.1.1    tibble_3.1.6        fansi_1.0.2
## [40] withr_2.4.3         crayon_1.4.2        dplyr_1.0.7
## [43] later_1.3.0         bitops_1.0-7        grid_4.1.2
## [46] nlme_3.1-153        jsonlite_1.7.3      xtable_1.8-4
## [49] gtable_0.3.0        lifecycle_1.0.1     DBI_1.1.2
## [52] magrittr_2.0.2      scales_1.1.1        stringi_1.7.6
## [55] cli_3.1.1           cachem_1.0.6        shinyMobile_0.9.1
## [58] farver_2.1.0        fs_1.5.2            promises_1.2.0.1
## [61] bslib_0.3.1         ellipsis_0.3.2      shinyFiles_0.9.1
## [64] generics_0.1.1      vctrs_0.3.8         EBImage_4.36.0
## [67] tools_4.1.2         glue_1.6.1          purrr_0.3.4
## [70] crosstalk_1.2.0     jpeg_0.1-9          abind_1.4-5
## [73] fastmap_1.1.0       yaml_2.2.2          colorspace_2.0-2
## [76] knitr_1.36          sass_0.4.0
```

Calibration Analysis

18 February 2022

Contents

1 Import of Data 1

2 Model 2

3 Analysis 2

4 Computation of LOB, LOD and LOQ 3

5 Save Results 4

6 Save Model 4

7 Software 4

1 Import of Data

We import the data and select the variables needed for the analysis.

```
load(file.path(PATH.OUT, paste0(FILENAME, "_Data.RData")))
tmp <- strsplit(FORMULA, "~")[[1]]
y.var <- names(unlist(sapply(colnames(CalibrationData),
                             grep, x = tmp[1])))
x.vars <- names(unlist(sapply(colnames(CalibrationData),
                              grep, x = tmp[2])))
if(SUBSET != ""){
  calData <- eval(call("subset", x =  CalibrationData,
                       subset = parse(text = SUBSET)))
}else{
  calData <- CalibrationData
}
calData <- calData[,c(y.var, x.vars)]
calData
```

```
##         Mean1      Mean2 conc
## 1  0.02173602 0.09050628    0
## 2  0.02462270 0.08726543    1
## 10 0.02084714 0.08911518    5
## 3  0.02714232 0.07805353   10
## 5  0.03076193 0.08199448   15
## 6  0.03337865 0.07498749   20
## 7  0.03674194 0.07210627   25
## 8  0.04009818 0.06788850   30
## 9  0.05087628 0.05783383   40
## 11 0.05878037 0.04570873   60
```

```
## 12 0.06536665 0.04013061   80
## 4  0.06764732 0.04109463  100
```

2 Model

We will apply the following model.

```
FORMULA
```

```
## [1] "(Mean1 / Mean2) ~ conc"
```

3 Analysis

We now fit (simple) linear model.

```
fit <- lm(as.formula(FORMULA), data = calData)
summary(fit)
```

```
##
## Call:
## lm(formula = as.formula(FORMULA), data = calData)
##
## Residuals:
##      Min       1Q   Median       3Q      Max
## -0.15371 -0.06796 -0.01662  0.06017  0.15168
##
## Coefficients:
##             Estimate Std. Error t value Pr(>|t|)
## (Intercept) 0.1864529  0.0408863    4.56  0.00104 **
## conc        0.0161340  0.0009166   17.60 7.45e-09 ***
## ---
## Signif. codes:  0 '***' 0.001 '**' 0.01 '*' 0.05 '.' 0.1 ' ' 1
##
## Residual standard error: 0.09812 on 10 degrees of freedom
## Multiple R-squared:  0.9687, Adjusted R-squared:  0.9656
## F-statistic: 309.8 on 1 and 10 DF,  p-value: 7.453e-09
```

We determine the inverse of the fitted model.

```
ab <- coef(fit)
names(ab) <- NULL
predFunc <- function(newdata){}
body(predFunc) <- substitute({ with(newdata, (eval(y)-a)/b) },
                             list(y = parse(text = respVar),
                                a = ab[1],
                                b = ab[2]))
```

We plot the given concentrations against the fitted values.

```
library(ggplot2)
modelPlot <- ggplot(calData, aes_string(x = concVar, y = respVar)) +
            geom_point() + geom_smooth(method = "lm") +
            annotate("text",  x=-Inf, y = Inf,
            label = substitute(paste(R^2, " = ", R2, ", adj. ", R^2, " = ", adj.R2),
                               list(R2 = signif(summary(fit)$r.squared,3),
                                    adj.R2 = signif(summary(fit)$adj.r.squared, 3))),
```

```
              vjust=1, hjust=0, size = 5)
modelPlot
```

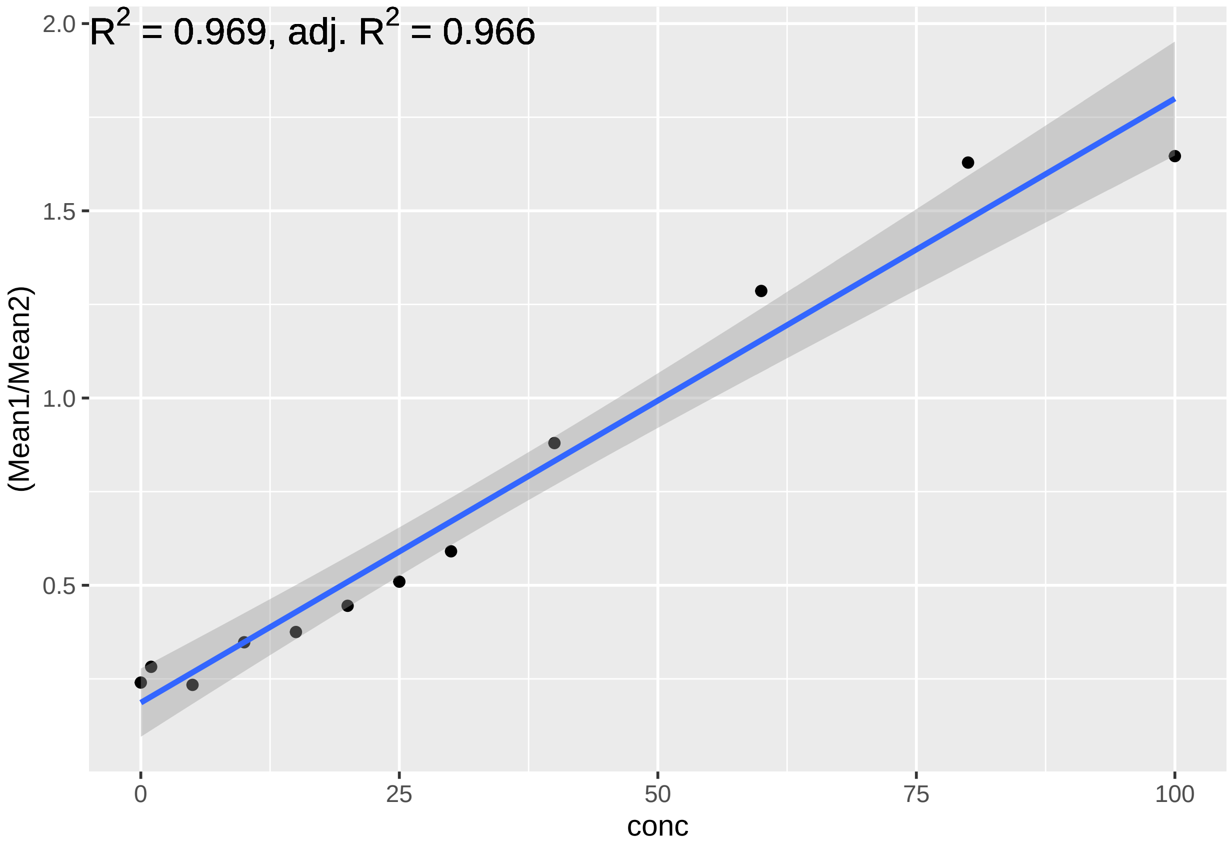

4 Computation of LOB, LOD and LOQ

We compute limit of blank (LOB), limit of detection (LOD) and limit of quantification (LOQ) by inverting the regression fit. We get the LOB by inverting the upper bound of the one-sided 95% confidence interval at concentration 0. In case of LOD, the upper-bound of the 99.95% confidence interval at concentration 0 is inverted. LOQ is determined as $3 \times$ LOD.

```
if(ab[2] > 0){
  LOB <- (confint(fit, parm = 1, level = 0.90)[2]-ab[1])/ab[2]
  LOD <- (confint(fit, parm = 1, level = 0.999)[2]-ab[1])/ab[2]
}else{
  LOB <- (confint(fit, parm = 1, level = 0.90)[1]-ab[1])/ab[2]
  LOD <- (confint(fit, parm = 1, level = 0.999)[1]-ab[1])/ab[2]
}
names(LOB) <- "LOB"
names(LOD) <- "LOD"
LOQ <- 3*LOD
names(LOQ) <- "LOQ"
LOB
```

```
##      LOB
## 4.593093
```

```
LOD
```

```
##      LOD
## 11.62399
```

```
LOQ
```

```
##      LOQ
## 34.87197
```

5 Save Results

We save the results.

```
save(fit, LOB, LOD, LOQ, file = file.path(PATH.OUT, paste0(FILENAME, "_Results.RData")))
```

6 Save Model

We save the inverse of the fitted model to be able to apply it for predicting concentrations.

```
saveRDS(object = predFunc, file = file.path(PATH.OUT, paste0(FILENAME, "_Model.rds")))
```

7 Software

```
sessionInfo()
```

```
## R version 4.1.2 (2021-11-01)
## Platform: x86_64-pc-linux-gnu (64-bit)
## Running under: Arch Linux
##
## Matrix products: default
## BLAS:   /usr/lib/libopenblasp-r0.3.19.so
## LAPACK: /usr/lib/liblapack.so.3.10.0
##
## locale:
##  [1] LC_CTYPE=en_US.UTF-8       LC_NUMERIC=C
##  [3] LC_TIME=en_US.UTF-8        LC_COLLATE=en_US.UTF-8
##  [5] LC_MONETARY=en_US.UTF-8    LC_MESSAGES=en_US.UTF-8
##  [7] LC_PAPER=en_US.UTF-8       LC_NAME=C
##  [9] LC_ADDRESS=C               LC_TELEPHONE=C
## [11] LC_MEASUREMENT=en_US.UTF-8 LC_IDENTIFICATION=C
##
## attached base packages:
## [1] stats     graphics  grDevices utils     datasets  methods   base
##
## other attached packages:
## [1] ggplot2_3.3.5 shiny_1.7.1
##
## loaded via a namespace (and not attached):
##  [1] Rcpp_1.0.8          locfit_1.5-9.4      lattice_0.20-45
##  [4] fftwtools_0.9-11    png_0.1-7           assertthat_0.2.1
##  [7] digest_0.6.29       utf8_1.2.2          mime_0.12
## [10] R6_2.5.1            tiff_0.1-10         evaluate_0.14
```

```
## [13] highr_0.9           pillar_1.7.0        rlang_1.0.1
## [16] fontawesome_0.2.2   jquerylib_0.1.4     Matrix_1.3-4
## [19] DT_0.20             rmarkdown_2.11      labeling_0.4.2
## [22] shinythemes_1.2.0   splines_4.1.2       shinyjs_2.1.0
## [25] LFApp_1.3           stringr_1.4.0       htmlwidgets_1.5.4
## [28] tinytex_0.35        RCurl_1.98-1.5      munsell_0.5.0
## [31] xfun_0.29           compiler_4.1.2      httpuv_1.6.5
## [34] pkgconfig_2.0.3     BiocGenerics_0.40.0 mgcv_1.8-38
## [37] htmltools_0.5.2     tidyselect_1.1.1    tibble_3.1.6
## [40] fansi_1.0.2         withr_2.4.3         crayon_1.4.2
## [43] dplyr_1.0.7         later_1.3.0         bitops_1.0-7
## [46] grid_4.1.2          nlme_3.1-153        jsonlite_1.7.3
## [49] xtable_1.8-4        gtable_0.3.0        lifecycle_1.0.1
## [52] DBI_1.1.2           magrittr_2.0.2      scales_1.1.1
## [55] stringi_1.7.6       cli_3.1.1           cachem_1.0.6
## [58] shinyMobile_0.9.1   farver_2.1.0        fs_1.5.2
## [61] promises_1.2.0.1    bslib_0.3.1         ellipsis_0.3.2
## [64] shinyFiles_0.9.1    generics_0.1.1      vctrs_0.3.8
## [67] EBImage_4.36.0      tools_4.1.2         glue_1.6.1
## [70] purrr_0.3.4         crosstalk_1.2.0     jpeg_0.1-9
## [73] abind_1.4-5         fastmap_1.1.0       yaml_2.2.2
## [76] colorspace_2.0-2    knitr_1.36          sass_0.4.0
```

Calibration Analysis

18 February 2022

Contents

1 **Import of Data** 1

2 **Model** 2

3 **Analysis** 2

4 **Computation of LOB, LOD and LOQ** 3

5 **Save Results** 4

6 **Save Model** 4

7 **Software** 4

1 Import of Data

We import the data and select the variables needed for the analysis.

```
load(file.path(PATH.OUT, paste0(FILENAME, "_Data.RData")))
tmp <- strsplit(FORMULA, "~")[[1]]
y.var <- names(unlist(sapply(colnames(CalibrationData),
                             grep, x = tmp[1])))
x.vars <- names(unlist(sapply(colnames(CalibrationData),
                              grep, x = tmp[2])))
if(SUBSET != ""){
  calData <- eval(call("subset", x =  CalibrationData,
                       subset = parse(text = SUBSET)))
}else{
  calData <- CalibrationData
}
calData <- calData[,c(y.var, x.vars)]
calData
```

```
##        Mean1      Mean2 conc
## 7 0.01548952 0.10201792    0
## 1 0.01650187 0.10398180    1
## 3 0.03249849 0.08843847   20
## 4 0.07038265 0.07916764   40
## 5 0.08973441 0.06737851   60
## 6 0.09333523 0.06038921   80
## 2 0.09289425 0.04603273  100
```

2 Model

We will apply the following model.

```
FORMULA
```

```
## [1] "(Mean1 / Mean2) ~ conc"
```

3 Analysis

We now fit (simple) linear model.

```
fit <- lm(as.formula(FORMULA), data = calData)
summary(fit)
```

```
##
## Call:
## lm(formula = as.formula(FORMULA), data = calData)
##
## Residuals:
##        7        1        3        4        5        6        2
##  0.03821  0.02626 -0.12270  0.02232  0.08853 -0.07426  0.02164
##
## Coefficients:
##              Estimate Std. Error t value Pr(>|t|)
## (Intercept) 0.1136168  0.0467709   2.429   0.0594 .
## conc        0.0188275  0.0008343  22.568 3.18e-06 ***
## ---
## Signif. codes:  0 '***' 0.001 '**' 0.01 '*' 0.05 '.' 0.1 ' ' 1
##
## Residual standard error: 0.0794 on 5 degrees of freedom
## Multiple R-squared:  0.9903, Adjusted R-squared:  0.9883
## F-statistic: 509.3 on 1 and 5 DF,  p-value: 3.175e-06
```

We determine the inverse of the fitted model.

```
ab <- coef(fit)
names(ab) <- NULL
predFunc <- function(newdata){}
body(predFunc) <- substitute({ with(newdata, (eval(y)-a)/b) },
                             list(y = parse(text = respVar),
                                a = ab[1],
                                b = ab[2]))
```

We plot the given concentrations against the fitted values.

```
library(ggplot2)
modelPlot <- ggplot(calData, aes_string(x = concVar, y = respVar)) +
            geom_point() + geom_smooth(method = "lm") +
            annotate("text",  x=-Inf, y = Inf,
            label = substitute(paste(R^2, " = ", R2, ", adj. ", R^2, " = ", adj.R2),
                              list(R2 = signif(summary(fit)$r.squared,3),
                                   adj.R2 = signif(summary(fit)$adj.r.squared, 3))),
            vjust=1, hjust=0, size = 5)
modelPlot
```

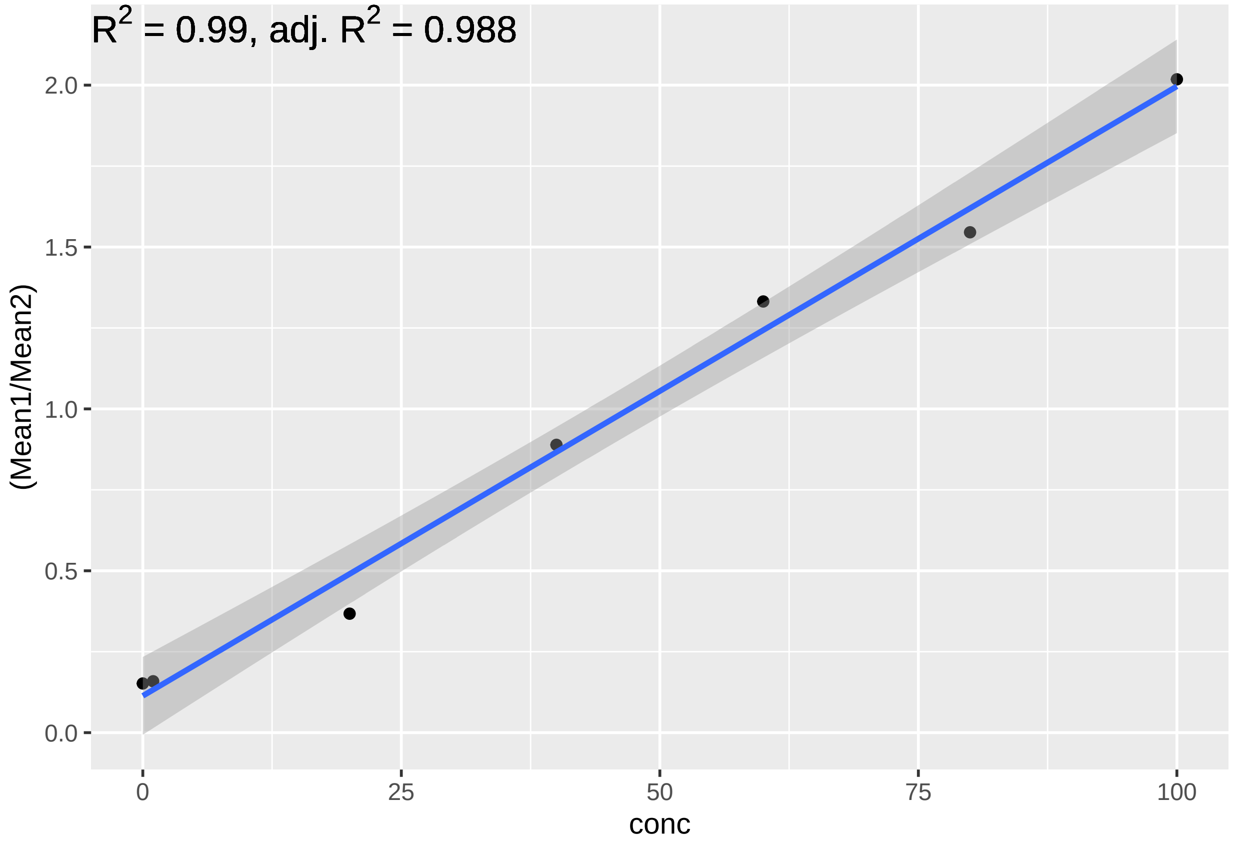

4 Computation of LOB, LOD and LOQ

We compute limit of blank (LOB), limit of detection (LOD) and limit of quantification (LOQ) by inverting the regression fit. We get the LOB by inverting the upper bound of the one-sided 95% confidence interval at concentration 0. In case of LOD, the upper-bound of the 99.95% confidence interval at concentration 0 is inverted. LOQ is determined as $3 \times$ LOD.

```
if(ab[2] > 0){
  LOB <- (confint(fit, parm = 1, level = 0.90)[2]-ab[1])/ab[2]
  LOD <- (confint(fit, parm = 1, level = 0.999)[2]-ab[1])/ab[2]
}else{
  LOB <- (confint(fit, parm = 1, level = 0.90)[1]-ab[1])/ab[2]
  LOD <- (confint(fit, parm = 1, level = 0.999)[1]-ab[1])/ab[2]
}
names(LOB) <- "LOB"
names(LOD) <- "LOD"
LOQ <- 3*LOD
names(LOQ) <- "LOQ"
LOB
```

```
##     LOB
## 5.00574
```

```
LOD
```

```
##      LOD
## 17.06339
```

```r
LOQ
```

```
##      LOQ
## 51.19017
```

5 Save Results

We save the results.

```r
save(fit, LOB, LOD, LOQ, file = file.path(PATH.OUT, paste0(FILENAME, "_Results.RData")))
```

6 Save Model

We save the inverse of the fitted model to be able to apply it for predicting concentrations.

```r
saveRDS(object = predFunc, file = file.path(PATH.OUT, paste0(FILENAME, "_Model.rds")))
```

7 Software

```r
sessionInfo()
```

```
## R version 4.1.2 (2021-11-01)
## Platform: x86_64-pc-linux-gnu (64-bit)
## Running under: Arch Linux
##
## Matrix products: default
## BLAS:   /usr/lib/libopenblasp-r0.3.19.so
## LAPACK: /usr/lib/liblapack.so.3.10.0
##
## locale:
##  [1] LC_CTYPE=en_US.UTF-8       LC_NUMERIC=C
##  [3] LC_TIME=en_US.UTF-8        LC_COLLATE=en_US.UTF-8
##  [5] LC_MONETARY=en_US.UTF-8    LC_MESSAGES=en_US.UTF-8
##  [7] LC_PAPER=en_US.UTF-8       LC_NAME=C
##  [9] LC_ADDRESS=C               LC_TELEPHONE=C
## [11] LC_MEASUREMENT=en_US.UTF-8 LC_IDENTIFICATION=C
##
## attached base packages:
## [1] stats     graphics  grDevices utils     datasets  methods   base
##
## other attached packages:
## [1] ggplot2_3.3.5 shiny_1.7.1
##
## loaded via a namespace (and not attached):
##  [1] Rcpp_1.0.8         locfit_1.5-9.4     lattice_0.20-45
##  [4] fftwtools_0.9-11   png_0.1-7          assertthat_0.2.1
##  [7] digest_0.6.29      utf8_1.2.2         mime_0.12
## [10] R6_2.5.1           tiff_0.1-10        evaluate_0.14
## [13] highr_0.9          pillar_1.7.0       rlang_1.0.1
## [16] fontawesome_0.2.2  jquerylib_0.1.4    Matrix_1.3-4
## [19] DT_0.20            rmarkdown_2.11     labeling_0.4.2
## [22] shinythemes_1.2.0  splines_4.1.2      shinyjs_2.1.0
```

```
## [25] LFApp_1.3           stringr_1.4.0       htmlwidgets_1.5.4
## [28] tinytex_0.35        RCurl_1.98-1.5      munsell_0.5.0
## [31] xfun_0.29           compiler_4.1.2      httpuv_1.6.5
## [34] pkgconfig_2.0.3     BiocGenerics_0.40.0 mgcv_1.8-38
## [37] htmltools_0.5.2     tidyselect_1.1.1    tibble_3.1.6
## [40] fansi_1.0.2         withr_2.4.3         crayon_1.4.2
## [43] dplyr_1.0.7         later_1.3.0         bitops_1.0-7
## [46] grid_4.1.2          nlme_3.1-153        jsonlite_1.7.3
## [49] xtable_1.8-4        gtable_0.3.0        lifecycle_1.0.1
## [52] DBI_1.1.2           magrittr_2.0.2      scales_1.1.1
## [55] stringi_1.7.6       cli_3.1.1           cachem_1.0.6
## [58] shinyMobile_0.9.1   farver_2.1.0        fs_1.5.2
## [61] promises_1.2.0.1    bslib_0.3.1         ellipsis_0.3.2
## [64] shinyFiles_0.9.1    generics_0.1.1      vctrs_0.3.8
## [67] EBImage_4.36.0      tools_4.1.2         glue_1.6.1
## [70] purrr_0.3.4         crosstalk_1.2.0     jpeg_0.1-9
## [73] abind_1.4-5         fastmap_1.1.0       yaml_2.2.2
## [76] colorspace_2.0-2    knitr_1.36          sass_0.4.0
```

Calibration Analysis

18 February 2022

Contents

1 Import of Data 1

2 Model 2

3 Analysis 2

4 Computation of LOB, LOD and LOQ 3

5 Save Results 4

6 Save Model 4

7 Software 4

1 Import of Data

We import the data and select the variables needed for the analysis.

```
load(file.path(PATH.OUT, paste0(FILENAME, "_Data.RData")))
tmp <- strsplit(FORMULA, "~")[[1]]
y.var <- names(unlist(sapply(colnames(CalibrationData),
                             grep, x = tmp[1])))
x.vars <- names(unlist(sapply(colnames(CalibrationData),
                              grep, x = tmp[2])))
if(SUBSET != ""){
  calData <- eval(call("subset", x =  CalibrationData,
                       subset = parse(text = SUBSET)))
}else{
  calData <- CalibrationData
}
calData <- calData[,c(y.var, x.vars)]
calData
```

```
##         Mean1      Mean2 conc
## 1  0.03977546 0.09425355    0
## 2  0.04466377 0.09182495    1
## 10 0.03545000 0.09155242    5
## 3  0.04859929 0.08681647   10
## 5  0.05339840 0.08678604   15
## 6  0.05817284 0.08363419   20
## 7  0.06218433 0.07842884   25
## 8  0.06649310 0.08182247   30
## 9  0.07823658 0.08575067   40
## 11 0.08815747 0.07859045   60
```

```
## 12 0.09344426 0.07149878   80
## 4  0.09451463 0.06011263  100
```

2 Model

We will apply the following model.

```
FORMULA
```

```
## [1] "(Mean1 / Mean2) ~ conc"
```

3 Analysis

We now fit (simple) linear model.

```
fit <- lm(as.formula(FORMULA), data = calData)
summary(fit)
```

```
##
## Call:
## lm(formula = as.formula(FORMULA), data = calData)
##
## Residuals:
##       Min        1Q    Median        3Q       Max
## -0.113106 -0.006064  0.002531  0.026902  0.066712
##
## Coefficients:
##             Estimate Std. Error t value Pr(>|t|)
## (Intercept)  0.44385    0.01963   22.61 6.44e-10 ***
## conc         0.01129    0.00044   25.66 1.85e-10 ***
## ---
## Signif. codes:  0 '***' 0.001 '**' 0.01 '*' 0.05 '.' 0.1 ' ' 1
##
## Residual standard error: 0.04711 on 10 degrees of freedom
## Multiple R-squared:  0.985,  Adjusted R-squared:  0.9835
## F-statistic: 658.5 on 1 and 10 DF,  p-value: 1.854e-10
```

We determine the inverse of the fitted model.

```
ab <- coef(fit)
names(ab) <- NULL
predFunc <- function(newdata){}
body(predFunc) <- substitute({ with(newdata, (eval(y)-a)/b) },
                             list(y = parse(text = respVar),
                                a = ab[1],
                                b = ab[2]))
```

We plot the given concentrations against the fitted values.

```
library(ggplot2)
modelPlot <- ggplot(calData, aes_string(x = concVar, y = respVar)) +
            geom_point() + geom_smooth(method = "lm") +
            annotate("text",  x=-Inf, y = Inf,
            label = substitute(paste(R^2, " = ", R2, ", adj. ", R^2, " = ", adj.R2),
                              list(R2 = signif(summary(fit)$r.squared,3),
                                   adj.R2 = signif(summary(fit)$adj.r.squared, 3))),
```

```
            vjust=1, hjust=0, size = 5)
modelPlot
```

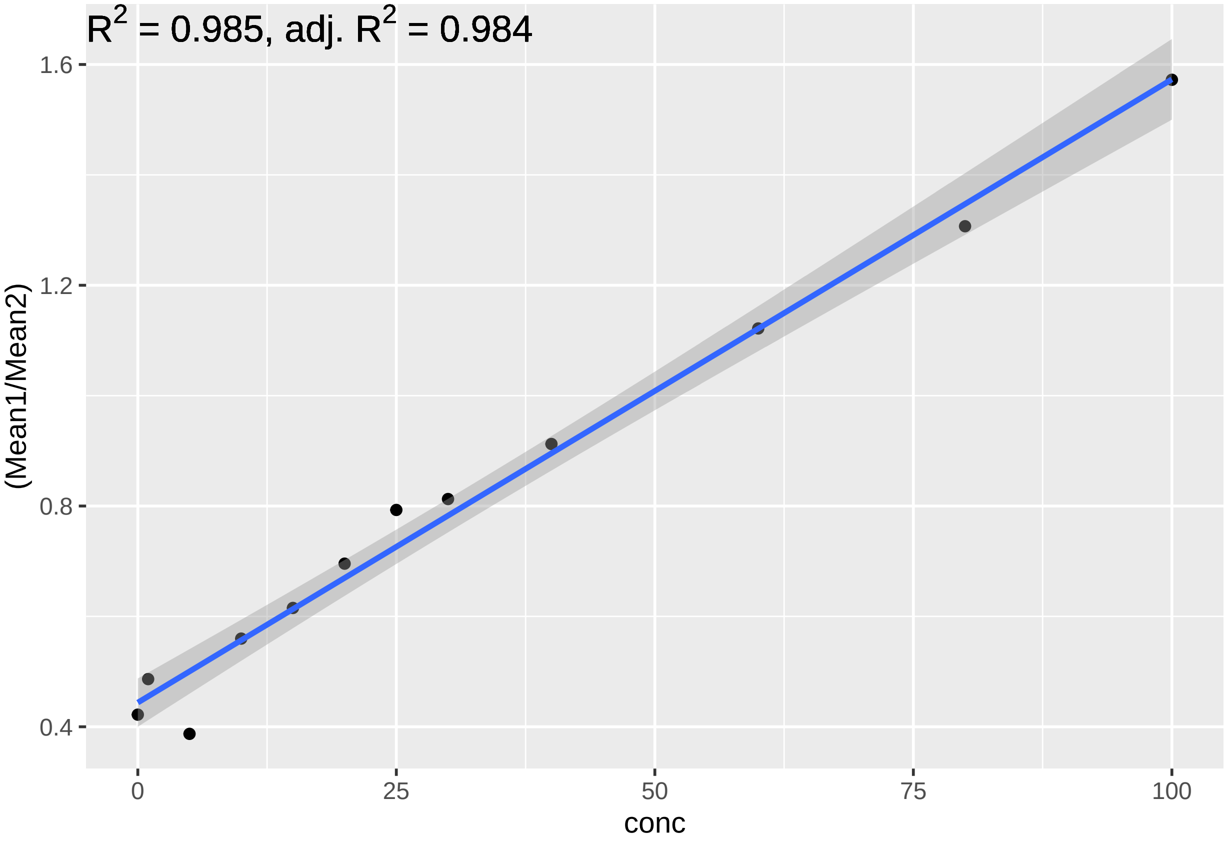

4 Computation of LOB, LOD and LOQ

We compute limit of blank (LOB), limit of detection (LOD) and limit of quantification (LOQ) by inverting the regression fit. We get the LOB by inverting the upper bound of the one-sided 95% confidence interval at concentration 0. In case of LOD, the upper-bound of the 99.95% confidence interval at concentration 0 is inverted. LOQ is determined as $3 \times$ LOD.

```
if(ab[2] > 0){
  LOB <- (confint(fit, parm = 1, level = 0.90)[2]-ab[1])/ab[2]
  LOD <- (confint(fit, parm = 1, level = 0.999)[2]-ab[1])/ab[2]
}else{
  LOB <- (confint(fit, parm = 1, level = 0.90)[1]-ab[1])/ab[2]
  LOD <- (confint(fit, parm = 1, level = 0.999)[1]-ab[1])/ab[2]
}
names(LOB) <- "LOB"
names(LOD) <- "LOD"
LOQ <- 3*LOD
names(LOQ) <- "LOQ"
LOB
```

```
##      LOB
## 3.150396
```

```r
LOD
```

```
##      LOD
## 7.972879
```

```r
LOQ
```

```
##      LOQ
## 23.91864
```

5 Save Results

We save the results.

```r
save(fit, LOB, LOD, LOQ, file = file.path(PATH.OUT, paste0(FILENAME, "_Results.RData")))
```

6 Save Model

We save the inverse of the fitted model to be able to apply it for predicting concentrations.

```r
saveRDS(object = predFunc, file = file.path(PATH.OUT, paste0(FILENAME, "_Model.rds")))
```

7 Software

```r
sessionInfo()
```

```
## R version 4.1.2 (2021-11-01)
## Platform: x86_64-pc-linux-gnu (64-bit)
## Running under: Arch Linux
##
## Matrix products: default
## BLAS:   /usr/lib/libopenblasp-r0.3.19.so
## LAPACK: /usr/lib/liblapack.so.3.10.0
##
## locale:
##  [1] LC_CTYPE=en_US.UTF-8       LC_NUMERIC=C
##  [3] LC_TIME=en_US.UTF-8        LC_COLLATE=en_US.UTF-8
##  [5] LC_MONETARY=en_US.UTF-8    LC_MESSAGES=en_US.UTF-8
##  [7] LC_PAPER=en_US.UTF-8       LC_NAME=C
##  [9] LC_ADDRESS=C               LC_TELEPHONE=C
## [11] LC_MEASUREMENT=en_US.UTF-8 LC_IDENTIFICATION=C
##
## attached base packages:
## [1] stats     graphics  grDevices utils     datasets  methods   base
##
## other attached packages:
## [1] ggplot2_3.3.5 shiny_1.7.1
##
## loaded via a namespace (and not attached):
##  [1] Rcpp_1.0.8          locfit_1.5-9.4      lattice_0.20-45
##  [4] fftwtools_0.9-11    png_0.1-7           assertthat_0.2.1
##  [7] digest_0.6.29       utf8_1.2.2          mime_0.12
## [10] R6_2.5.1            tiff_0.1-10         evaluate_0.14
```

```
## [13] highr_0.9           pillar_1.7.0        rlang_1.0.1
## [16] fontawesome_0.2.2   jquerylib_0.1.4     Matrix_1.3-4
## [19] DT_0.20             rmarkdown_2.11      labeling_0.4.2
## [22] shinythemes_1.2.0   splines_4.1.2       shinyjs_2.1.0
## [25] LFApp_1.3           stringr_1.4.0       htmlwidgets_1.5.4
## [28] tinytex_0.35        RCurl_1.98-1.5      munsell_0.5.0
## [31] xfun_0.29           compiler_4.1.2      httpuv_1.6.5
## [34] pkgconfig_2.0.3     BiocGenerics_0.40.0 mgcv_1.8-38
## [37] htmltools_0.5.2     tidyselect_1.1.1    tibble_3.1.6
## [40] fansi_1.0.2         withr_2.4.3         crayon_1.4.2
## [43] dplyr_1.0.7         later_1.3.0         bitops_1.0-7
## [46] grid_4.1.2          nlme_3.1-153        jsonlite_1.7.3
## [49] xtable_1.8-4        gtable_0.3.0        lifecycle_1.0.1
## [52] DBI_1.1.2           magrittr_2.0.2      scales_1.1.1
## [55] stringi_1.7.6       cli_3.1.1           cachem_1.0.6
## [58] shinyMobile_0.9.1   farver_2.1.0        fs_1.5.2
## [61] promises_1.2.0.1    bslib_0.3.1         ellipsis_0.3.2
## [64] shinyFiles_0.9.1    generics_0.1.1      vctrs_0.3.8
## [67] EBImage_4.36.0      tools_4.1.2         glue_1.6.1
## [70] purrr_0.3.4         crosstalk_1.2.0     jpeg_0.1-9
## [73] abind_1.4-5         fastmap_1.1.0       yaml_2.2.2
## [76] colorspace_2.0-2    knitr_1.36          sass_0.4.0
```

Calibration Analysis

18 February 2022

Contents

1 Import of Data 1

2 Model 2

3 Analysis 2

4 Computation of LOB, LOD and LOQ 3

5 Save Results 4

6 Save Model 4

7 Software 4

1 Import of Data

We import the data and select the variables needed for the analysis.

```
load(file.path(PATH.OUT, paste0(FILENAME, "_Data.RData")))
tmp <- strsplit(FORMULA, "~")[[1]]
y.var <- names(unlist(sapply(colnames(CalibrationData),
                              grep, x = tmp[1])))
x.vars <- names(unlist(sapply(colnames(CalibrationData),
                               grep, x = tmp[2])))
if(SUBSET != ""){
  calData <- eval(call("subset", x =  CalibrationData,
                       subset = parse(text = SUBSET)))
}else{
  calData <- CalibrationData
}
calData <- calData[,c(y.var, x.vars)]
calData
```

```
##         Mean2 thrombin
## 1 0.003936601     0.00
## 2 0.014649340     8.60
## 3 0.011499815     2.15
## 4 0.021068307    17.20
## 5 0.034727700    25.80
## 6 0.009374197     4.30
```

2 Model

We will apply the following model.

```
FORMULA
```

```
## [1] "(Mean2) ~ thrombin"
```

3 Analysis

We now fit (simple) linear model.

```
fit <- lm(as.formula(FORMULA), data = calData)
summary(fit)
```

```
##
## Call:
## lm(formula = as.formula(FORMULA), data = calData)
##
## Residuals:
##          1          2          3          4          5          6
## -1.664e-03 -8.492e-05  3.616e-03 -2.800e-03  1.726e-03 -7.931e-04
##
## Coefficients:
##              Estimate Std. Error t value Pr(>|t|)
## (Intercept) 0.0056004  0.0015565   3.598 0.022800 *
## thrombin    0.0010621  0.0001172   9.063 0.000821 ***
## ---
## Signif. codes:  0 '***' 0.001 '**' 0.01 '*' 0.05 '.' 0.1 ' ' 1
##
## Residual standard error: 0.002612 on 4 degrees of freedom
## Multiple R-squared:  0.9536, Adjusted R-squared:  0.942
## F-statistic: 82.14 on 1 and 4 DF,  p-value: 0.0008215
```

We determine the inverse of the fitted model.

```
ab <- coef(fit)
names(ab) <- NULL
predFunc <- function(newdata){}
body(predFunc) <- substitute({ with(newdata, (eval(y)-a)/b) },
                             list(y = parse(text = respVar),
                                a = ab[1],
                                b = ab[2]))
```

We plot the given concentrations against the fitted values.

```
library(ggplot2)
modelPlot <- ggplot(calData, aes_string(x = concVar, y = respVar)) +
            geom_point() + geom_smooth(method = "lm") +
            annotate("text",  x=-Inf, y = Inf,
            label = substitute(paste(R^2, " = ", R2, ", adj. ", R^2, " = ", adj.R2),
                              list(R2 = signif(summary(fit)$r.squared,3),
                                   adj.R2 = signif(summary(fit)$adj.r.squared, 3))),
            vjust=1, hjust=0, size = 5)
modelPlot
```

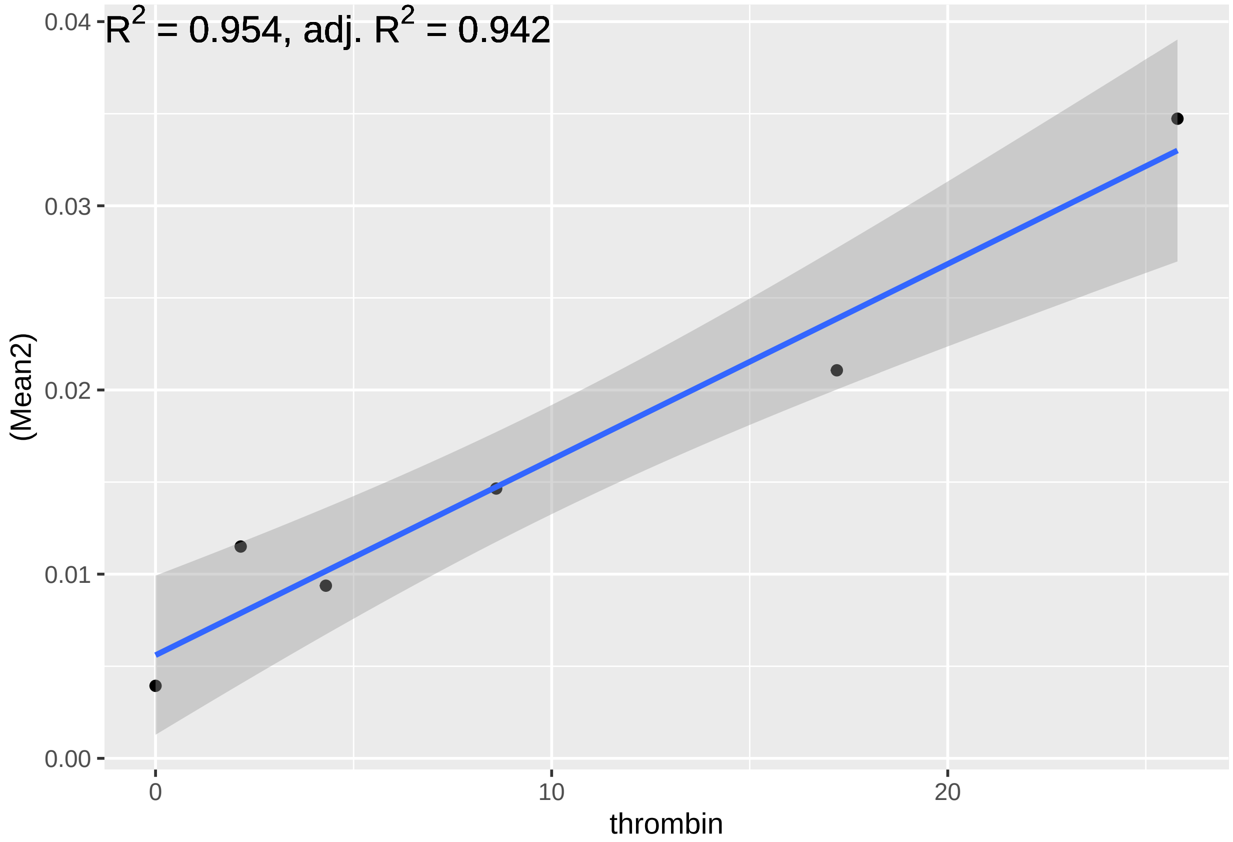

4 Computation of LOB, LOD and LOQ

We compute limit of blank (LOB), limit of detection (LOD) and limit of quantification (LOQ) by inverting the regression fit. We get the LOB by inverting the upper bound of the one-sided 95% confidence interval at concentration 0. In case of LOD, the upper-bound of the 99.95% confidence interval at concentration 0 is inverted. LOQ is determined as $3 \times$ LOD.

```
if(ab[2] > 0){
  LOB <- (confint(fit, parm = 1, level = 0.90)[2]-ab[1])/ab[2]
  LOD <- (confint(fit, parm = 1, level = 0.999)[2]-ab[1])/ab[2]
}else{
  LOB <- (confint(fit, parm = 1, level = 0.90)[1]-ab[1])/ab[2]
  LOD <- (confint(fit, parm = 1, level = 0.999)[1]-ab[1])/ab[2]
}
names(LOB) <- "LOB"
names(LOD) <- "LOD"
LOQ <- 3*LOD
names(LOQ) <- "LOQ"
LOB
```

```
##     LOB
## 3.12437
```

```
LOD
```

```
##    LOD
## 12.619
```

```
LOQ
```

```
##      LOQ
## 37.85699
```

5 Save Results

We save the results.

```
save(fit, LOB, LOD, LOQ, file = file.path(PATH.OUT, paste0(FILENAME, "_Results.RData")))
```

6 Save Model

We save the inverse of the fitted model to be able to apply it for predicting concentrations.

```
saveRDS(object = predFunc, file = file.path(PATH.OUT, paste0(FILENAME, "_Model.rds")))
```

7 Software

```
sessionInfo()
```

```
## R version 4.1.2 (2021-11-01)
## Platform: x86_64-pc-linux-gnu (64-bit)
## Running under: Arch Linux
##
## Matrix products: default
## BLAS:   /usr/lib/libopenblasp-r0.3.19.so
## LAPACK: /usr/lib/liblapack.so.3.10.0
##
## locale:
##  [1] LC_CTYPE=en_US.UTF-8       LC_NUMERIC=C
##  [3] LC_TIME=en_US.UTF-8        LC_COLLATE=en_US.UTF-8
##  [5] LC_MONETARY=en_US.UTF-8    LC_MESSAGES=en_US.UTF-8
##  [7] LC_PAPER=en_US.UTF-8       LC_NAME=C
##  [9] LC_ADDRESS=C               LC_TELEPHONE=C
## [11] LC_MEASUREMENT=en_US.UTF-8 LC_IDENTIFICATION=C
##
## attached base packages:
## [1] stats     graphics  grDevices utils     datasets  methods   base
##
## other attached packages:
## [1] ggplot2_3.3.5 shiny_1.7.1
##
## loaded via a namespace (and not attached):
##  [1] Rcpp_1.0.8          locfit_1.5-9.4      lattice_0.20-45
##  [4] fftwtools_0.9-11    png_0.1-7           assertthat_0.2.1
##  [7] digest_0.6.29       utf8_1.2.2          mime_0.12
## [10] R6_2.5.1            tiff_0.1-10         evaluate_0.14
## [13] highr_0.9           pillar_1.7.0        rlang_1.0.1
## [16] fontawesome_0.2.2   jquerylib_0.1.4     Matrix_1.3-4
## [19] DT_0.20             rmarkdown_2.11      labeling_0.4.2
## [22] shinythemes_1.2.0   splines_4.1.2       shinyjs_2.1.0
```

```
## [25] LFApp_1.3           stringr_1.4.0       htmlwidgets_1.5.4
## [28] tinytex_0.35        RCurl_1.98-1.5      munsell_0.5.0
## [31] xfun_0.29           compiler_4.1.2      httpuv_1.6.5
## [34] pkgconfig_2.0.3     BiocGenerics_0.40.0 mgcv_1.8-38
## [37] htmltools_0.5.2     tidyselect_1.1.1    tibble_3.1.6
## [40] fansi_1.0.2         withr_2.4.3         crayon_1.4.2
## [43] dplyr_1.0.7         later_1.3.0         bitops_1.0-7
## [46] grid_4.1.2          nlme_3.1-153        jsonlite_1.7.3
## [49] xtable_1.8-4        gtable_0.3.0        lifecycle_1.0.1
## [52] DBI_1.1.2           magrittr_2.0.2      scales_1.1.1
## [55] stringi_1.7.6       cli_3.1.1           cachem_1.0.6
## [58] shinyMobile_0.9.1   farver_2.1.0        fs_1.5.2
## [61] promises_1.2.0.1    bslib_0.3.1         ellipsis_0.3.2
## [64] shinyFiles_0.9.1    generics_0.1.1      vctrs_0.3.8
## [67] EBImage_4.36.0      tools_4.1.2         glue_1.6.1
## [70] purrr_0.3.4         crosstalk_1.2.0     jpeg_0.1-9
## [73] abind_1.4-5         fastmap_1.1.0       yaml_2.2.2
## [76] colorspace_2.0-2    knitr_1.36          sass_0.4.0
```

Calibration Analysis

18 February 2022

Contents

1 Import of Data 1

2 Model 2

3 Analysis 2

4 Computation of LOB, LOD and LOQ 3

5 Save Results 4

6 Save Model 4

7 Software 4

1 Import of Data

We import the data and select the variables needed for the analysis.

```
load(file.path(PATH.OUT, paste0(FILENAME, "_Data.RData")))
tmp <- strsplit(FORMULA, "~")[[1]]
y.var <- names(unlist(sapply(colnames(CalibrationData),
                             grep, x = tmp[1])))
x.vars <- names(unlist(sapply(colnames(CalibrationData),
                              grep, x = tmp[2])))
if(SUBSET != ""){
  calData <- eval(call("subset", x =  CalibrationData,
                       subset = parse(text = SUBSET)))
}else{
  calData <- CalibrationData
}
calData <- calData[,c(y.var, x.vars)]
calData
```

```
##        Mean1       Mean2    IL6
## 1 0.05153348 0.003936601  0.000
## 2 0.06618089 0.014649340 25.000
## 3 0.05385849 0.011499815  0.625
## 4 0.05025827 0.021068307 50.000
## 5 0.05329297 0.034727700 75.000
## 6 0.06710419 0.009374197 12.500
```

2 Model

We will apply the following model.

```
FORMULA
```

```
## [1] "(Mean2 / Mean1) ~ IL6"
```

3 Analysis

We now fit (simple) linear model.

```
fit <- lm(as.formula(FORMULA), data = calData)
summary(fit)
```

```
##
## Call:
## lm(formula = as.formula(FORMULA), data = calData)
##
## Residuals:
##        1        2        3        4        5        6
## -0.02553 -0.05072  0.10734 -0.02303  0.03925 -0.04730
##
## Coefficients:
##             Estimate Std. Error t value Pr(>|t|)
## (Intercept) 0.101923   0.039768   2.563  0.06244 .
## IL6         0.006806   0.001032   6.594  0.00274 **
## ---
## Signif. codes:  0 '***' 0.001 '**' 0.01 '*' 0.05 '.' 0.1 ' ' 1
##
## Residual standard error: 0.06902 on 4 degrees of freedom
## Multiple R-squared:  0.9158, Adjusted R-squared:  0.8947
## F-statistic: 43.48 on 1 and 4 DF,  p-value: 0.00274
```

We determine the inverse of the fitted model.

```
ab <- coef(fit)
names(ab) <- NULL
predFunc <- function(newdata){}
body(predFunc) <- substitute({ with(newdata, (eval(y)-a)/b) },
                             list(y = parse(text = respVar),
                                a = ab[1],
                                b = ab[2]))
```

We plot the given concentrations against the fitted values.

```
library(ggplot2)
modelPlot <- ggplot(calData, aes_string(x = concVar, y = respVar)) +
            geom_point() + geom_smooth(method = "lm") +
            annotate("text",  x=-Inf, y = Inf,
            label = substitute(paste(R^2, " = ", R2, ", adj. ", R^2, " = ", adj.R2),
                                list(R2 = signif(summary(fit)$r.squared,3),
                                     adj.R2 = signif(summary(fit)$adj.r.squared, 3))),
            vjust=1, hjust=0, size = 5)
modelPlot
```

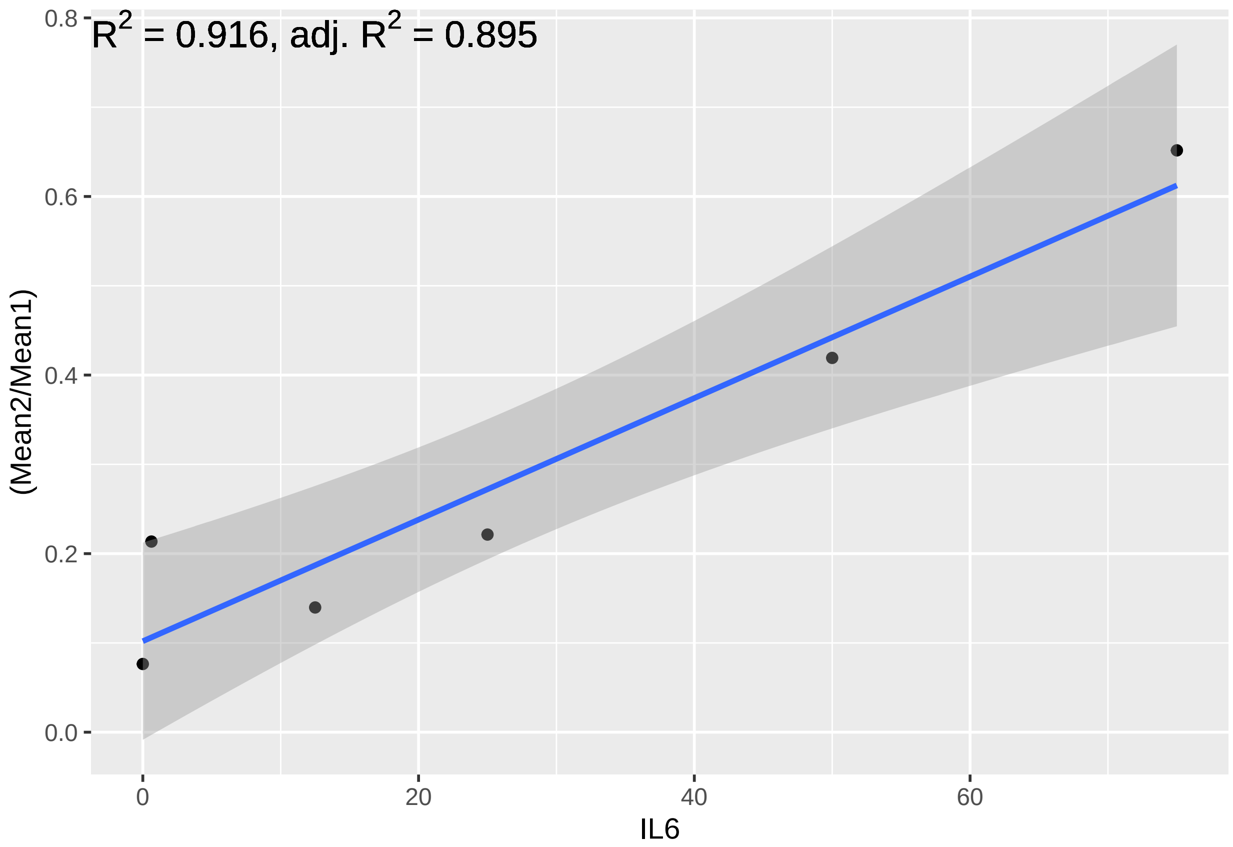

4 Computation of LOB, LOD and LOQ

We compute limit of blank (LOB), limit of detection (LOD) and limit of quantification (LOQ) by inverting the regression fit. We get the LOB by inverting the upper bound of the one-sided 95% confidence interval at concentration 0. In case of LOD, the upper-bound of the 99.95% confidence interval at concentration 0 is inverted. LOQ is determined as $3 \times$ LOD.

```
if(ab[2] > 0){
  LOB <- (confint(fit, parm = 1, level = 0.90)[2]-ab[1])/ab[2]
  LOD <- (confint(fit, parm = 1, level = 0.999)[2]-ab[1])/ab[2]
}else{
  LOB <- (confint(fit, parm = 1, level = 0.90)[1]-ab[1])/ab[2]
  LOD <- (confint(fit, parm = 1, level = 0.999)[1]-ab[1])/ab[2]
}
names(LOB) <- "LOB"
names(LOD) <- "LOD"
LOQ <- 3*LOD
names(LOQ) <- "LOQ"
LOB
```

```
##      LOB
## 12.45621
```

```
LOD
```

```
##      LOD
## 50.30929
```

```r
LOQ
```

```
##      LOQ
## 150.9279
```

5 Save Results

We save the results.

```r
save(fit, LOB, LOD, LOQ, file = file.path(PATH.OUT, paste0(FILENAME, "_Results.RData")))
```

6 Save Model

We save the inverse of the fitted model to be able to apply it for predicting concentrations.

```r
saveRDS(object = predFunc, file = file.path(PATH.OUT, paste0(FILENAME, "_Model.rds")))
```

7 Software

```r
sessionInfo()
```

```
## R version 4.1.2 (2021-11-01)
## Platform: x86_64-pc-linux-gnu (64-bit)
## Running under: Arch Linux
##
## Matrix products: default
## BLAS:   /usr/lib/libopenblasp-r0.3.19.so
## LAPACK: /usr/lib/liblapack.so.3.10.0
##
## locale:
##  [1] LC_CTYPE=en_US.UTF-8       LC_NUMERIC=C
##  [3] LC_TIME=en_US.UTF-8        LC_COLLATE=en_US.UTF-8
##  [5] LC_MONETARY=en_US.UTF-8    LC_MESSAGES=en_US.UTF-8
##  [7] LC_PAPER=en_US.UTF-8       LC_NAME=C
##  [9] LC_ADDRESS=C               LC_TELEPHONE=C
## [11] LC_MEASUREMENT=en_US.UTF-8 LC_IDENTIFICATION=C
##
## attached base packages:
## [1] stats     graphics  grDevices utils     datasets  methods   base
##
## other attached packages:
## [1] ggplot2_3.3.5 shiny_1.7.1
##
## loaded via a namespace (and not attached):
##  [1] Rcpp_1.0.8          locfit_1.5-9.4      lattice_0.20-45
##  [4] fftwtools_0.9-11    png_0.1-7           assertthat_0.2.1
##  [7] digest_0.6.29       utf8_1.2.2          mime_0.12
## [10] R6_2.5.1            tiff_0.1-10         evaluate_0.14
## [13] highr_0.9           pillar_1.7.0        rlang_1.0.1
## [16] fontawesome_0.2.2   jquerylib_0.1.4     Matrix_1.3-4
## [19] DT_0.20             rmarkdown_2.11      labeling_0.4.2
## [22] shinythemes_1.2.0   splines_4.1.2       shinyjs_2.1.0
```

```
## [25] LFApp_1.3           stringr_1.4.0       htmlwidgets_1.5.4
## [28] tinytex_0.35        RCurl_1.98-1.5      munsell_0.5.0
## [31] xfun_0.29           compiler_4.1.2      httpuv_1.6.5
## [34] pkgconfig_2.0.3     BiocGenerics_0.40.0 mgcv_1.8-38
## [37] htmltools_0.5.2     tidyselect_1.1.1    tibble_3.1.6
## [40] fansi_1.0.2         withr_2.4.3         crayon_1.4.2
## [43] dplyr_1.0.7         later_1.3.0         bitops_1.0-7
## [46] grid_4.1.2          nlme_3.1-153        jsonlite_1.7.3
## [49] xtable_1.8-4        gtable_0.3.0        lifecycle_1.0.1
## [52] DBI_1.1.2           magrittr_2.0.2      scales_1.1.1
## [55] stringi_1.7.6       cli_3.1.1           cachem_1.0.6
## [58] shinyMobile_0.9.1   farver_2.1.0        fs_1.5.2
## [61] promises_1.2.0.1    bslib_0.3.1         ellipsis_0.3.2
## [64] shinyFiles_0.9.1    generics_0.1.1      vctrs_0.3.8
## [67] EBImage_4.36.0      tools_4.1.2         glue_1.6.1
## [70] purrr_0.3.4         crosstalk_1.2.0     jpeg_0.1-9
## [73] abind_1.4-5         fastmap_1.1.0       yaml_2.2.2
## [76] colorspace_2.0-2    knitr_1.36          sass_0.4.0
```

Calibration Analysis

18 February 2022

Contents

1 Import of Data **1**

2 Model **2**

3 Analysis **2**

4 Computation of LOB, LOD and LOQ **3**

5 Save Results **4**

6 Save Model **4**

7 Software **4**

1 Import of Data

We import the data and select the variables needed for the analysis.

```
load(file.path(PATH.OUT, paste0(FILENAME, "_Data.RData")))
tmp <- strsplit(FORMULA, "~")[[1]]
y.var <- names(unlist(sapply(colnames(CalibrationData),
                             grep, x = tmp[1])))
x.vars <- names(unlist(sapply(colnames(CalibrationData),
                              grep, x = tmp[2])))
if(SUBSET != ""){
  calData <- eval(call("subset", x =  CalibrationData,
                       subset = parse(text = SUBSET)))
}else{
  calData <- CalibrationData
}
calData <- calData[,c(y.var, x.vars)]
calData
```

```
##    Mean1.green Mean2.green CRP
## 1  0.147551962 0.005841316   0
## 2  0.145517179 0.007713005   0
## 3  0.183435129 0.024305637   2
## 4  0.156022338 0.012892769   2
## 5  0.159285409 0.031339443   4
## 6  0.134223738 0.035040704   4
## 7  0.083343533 0.045234659   6
## 8  0.123443973 0.062582535   6
## 9  0.101518301 0.073029273   8
## 10 0.031960099 0.048549016   8
```

```
## 11 0.032337427 0.076519327  10
## 12 0.078928259 0.080070786  10
## 13 0.018166999 0.104067203  15
## 14 0.038219942 0.147207357  15
## 15 0.006650731 0.184773707  20
## 16 0.004402916 0.165787512  20
```

2 Model

We will apply the following model.

```
FORMULA
```

```
## [1] "(Mean2.green / (Mean1.green + Mean2.green)) ~ CRP"
```

3 Analysis

We now fit (simple) linear model.

```
fit <- lm(as.formula(FORMULA), data = calData)
summary(fit)
```

```
##
## Call:
## lm(formula = as.formula(FORMULA), data = calData)
##
## Residuals:
##       Min        1Q    Median        3Q       Max
## -0.077177 -0.043896 -0.013662  0.008694  0.162384
##
## Coefficients:
##             Estimate Std. Error t value Pr(>|t|)
## (Intercept) 0.042267   0.030908   1.368    0.193
## CRP         0.049829   0.003007  16.569 1.36e-10 ***
## ---
## Signif. codes:  0 '***' 0.001 '**' 0.01 '*' 0.05 '.' 0.1 ' ' 1
##
## Residual standard error: 0.07571 on 14 degrees of freedom
## Multiple R-squared:  0.9515, Adjusted R-squared:  0.948
## F-statistic: 274.5 on 1 and 14 DF,  p-value: 1.355e-10
```

We determine the inverse of the fitted model.

```
ab <- coef(fit)
names(ab) <- NULL
predFunc <- function(newdata){}
body(predFunc) <- substitute({ with(newdata, (eval(y)-a)/b) },
                             list(y = parse(text = respVar),
                                  a = ab[1],
                                  b = ab[2]))
```

We plot the given concentrations against the fitted values.

```
library(ggplot2)
modelPlot <- ggplot(calData, aes_string(x = concVar, y = respVar)) +
            geom_point() + geom_smooth(method = "lm") +
```

```
         annotate("text",  x=-Inf, y = Inf,
         label = substitute(paste(R^2, " = ", R2, ", adj. ", R^2, " = ", adj.R2),
                          list(R2 = signif(summary(fit)$r.squared,3),
                               adj.R2 = signif(summary(fit)$adj.r.squared, 3))),
         vjust=1, hjust=0, size = 5)
modelPlot
```

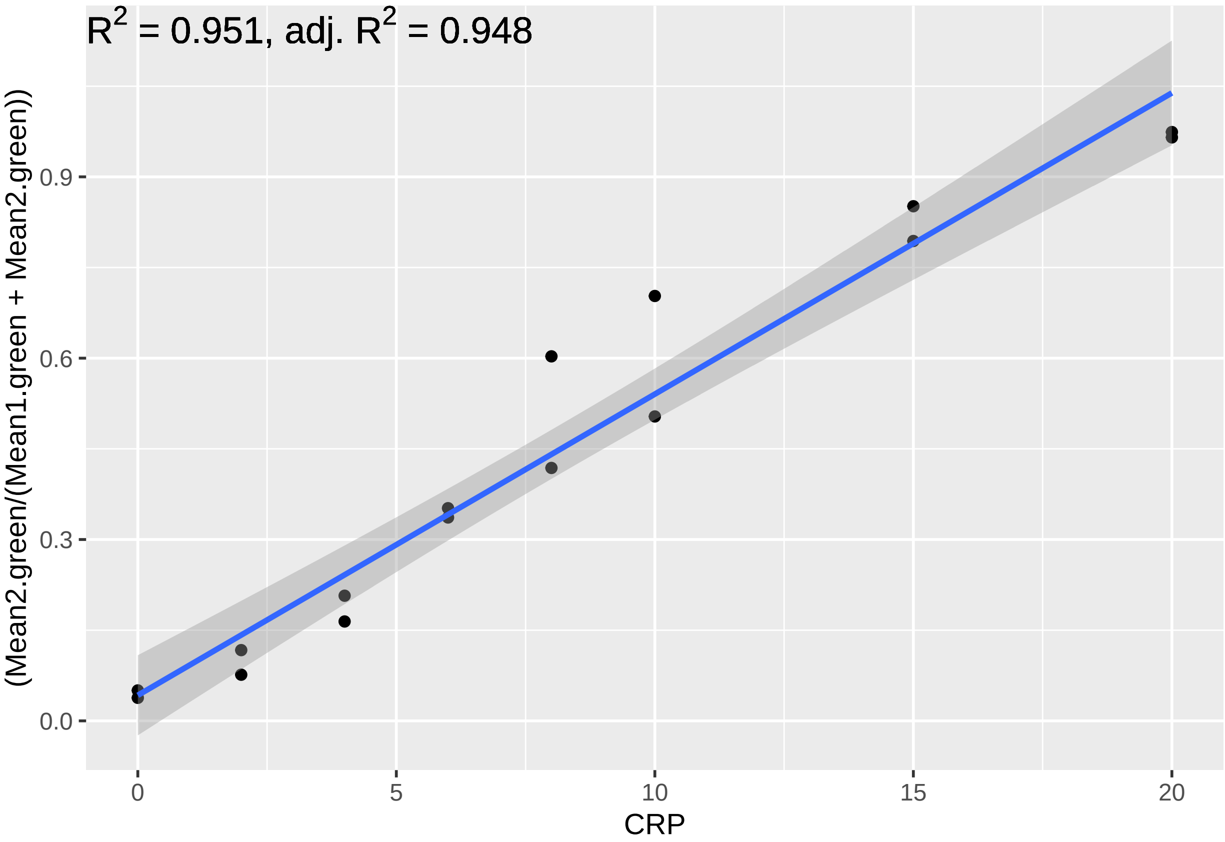

4 Computation of LOB, LOD and LOQ

We compute limit of blank (LOB), limit of detection (LOD) and limit of quantification (LOQ) by inverting the regression fit. We get the LOB by inverting the upper bound of the one-sided 95% confidence interval at concentration 0. In case of LOD, the upper-bound of the 99.95% confidence interval at concentration 0 is inverted. LOQ is determined as $3 \times$ LOD.

```
if(ab[2] > 0){
  LOB <- (confint(fit, parm = 1, level = 0.90)[2]-ab[1])/ab[2]
  LOD <- (confint(fit, parm = 1, level = 0.999)[2]-ab[1])/ab[2]
}else{
  LOB <- (confint(fit, parm = 1, level = 0.90)[1]-ab[1])/ab[2]
  LOD <- (confint(fit, parm = 1, level = 0.999)[1]-ab[1])/ab[2]
}
names(LOB) <- "LOB"
names(LOD) <- "LOD"
LOQ <- 3*LOD
names(LOQ) <- "LOQ"
LOB
```

```
##      LOB
## 1.092501
```

```r
LOD
```

```
##      LOD
## 2.568231
```

```r
LOQ
```

```
##      LOQ
## 7.704693
```

5 Save Results

We save the results.

```r
save(fit, LOB, LOD, LOQ, file = file.path(PATH.OUT, paste0(FILENAME, "_Results.RData")))
```

6 Save Model

We save the inverse of the fitted model to be able to apply it for predicting concentrations.

```r
saveRDS(object = predFunc, file = file.path(PATH.OUT, paste0(FILENAME, "_Model.rds")))
```

7 Software

```r
sessionInfo()
```

```
## R version 4.1.2 (2021-11-01)
## Platform: x86_64-pc-linux-gnu (64-bit)
## Running under: Arch Linux
##
## Matrix products: default
## BLAS:   /usr/lib/libopenblasp-r0.3.19.so
## LAPACK: /usr/lib/liblapack.so.3.10.0
##
## locale:
##  [1] LC_CTYPE=en_US.UTF-8       LC_NUMERIC=C
##  [3] LC_TIME=en_US.UTF-8        LC_COLLATE=en_US.UTF-8
##  [5] LC_MONETARY=en_US.UTF-8    LC_MESSAGES=en_US.UTF-8
##  [7] LC_PAPER=en_US.UTF-8       LC_NAME=C
##  [9] LC_ADDRESS=C               LC_TELEPHONE=C
## [11] LC_MEASUREMENT=en_US.UTF-8 LC_IDENTIFICATION=C
##
## attached base packages:
## [1] stats     graphics  grDevices utils     datasets  methods   base
##
## other attached packages:
## [1] ggplot2_3.3.5 shiny_1.7.1
##
## loaded via a namespace (and not attached):
##  [1] Rcpp_1.0.8          locfit_1.5-9.4      lattice_0.20-45
##  [4] fftwtools_0.9-11    png_0.1-7           assertthat_0.2.1
```

```
##  [7] digest_0.6.29       utf8_1.2.2          mime_0.12
## [10] R6_2.5.1            tiff_0.1-10         evaluate_0.14
## [13] highr_0.9           pillar_1.7.0        rlang_1.0.1
## [16] fontawesome_0.2.2   jquerylib_0.1.4     Matrix_1.3-4
## [19] DT_0.20             rmarkdown_2.11      labeling_0.4.2
## [22] shinythemes_1.2.0   splines_4.1.2       shinyjs_2.1.0
## [25] LFApp_1.3           stringr_1.4.0       htmlwidgets_1.5.4
## [28] tinytex_0.35        RCurl_1.98-1.5      munsell_0.5.0
## [31] xfun_0.29           compiler_4.1.2      httpuv_1.6.5
## [34] pkgconfig_2.0.3     BiocGenerics_0.40.0 mgcv_1.8-38
## [37] htmltools_0.5.2     tidyselect_1.1.1    tibble_3.1.6
## [40] fansi_1.0.2         withr_2.4.3         crayon_1.4.2
## [43] dplyr_1.0.7         later_1.3.0         bitops_1.0-7
## [46] grid_4.1.2          nlme_3.1-153        jsonlite_1.7.3
## [49] xtable_1.8-4        gtable_0.3.0        lifecycle_1.0.1
## [52] DBI_1.1.2           magrittr_2.0.2      scales_1.1.1
## [55] stringi_1.7.6       cli_3.1.1           cachem_1.0.6
## [58] shinyMobile_0.9.1   farver_2.1.0        fs_1.5.2
## [61] promises_1.2.0.1    bslib_0.3.1         ellipsis_0.3.2
## [64] shinyFiles_0.9.1    generics_0.1.1      vctrs_0.3.8
## [67] EBImage_4.36.0      tools_4.1.2         glue_1.6.1
## [70] purrr_0.3.4         crosstalk_1.2.0     jpeg_0.1-9
## [73] abind_1.4-5         fastmap_1.1.0       yaml_2.2.2
## [76] colorspace_2.0-2    knitr_1.36          sass_0.4.0
```

Calibration Analysis

18 February 2022

Contents

1 Import of Data **1**

2 Model **2**

3 Analysis **2**

4 Computation of LOB, LOD and LOQ **3**

5 Save Results **4**

6 Save Model **4**

7 Software **4**

1 Import of Data

We import the data and select the variables needed for the analysis.

```
load(file.path(PATH.OUT, paste0(FILENAME, "_Data.RData")))
tmp <- strsplit(FORMULA, "~")[[1]]
y.var <- names(unlist(sapply(colnames(CalibrationData),
                             grep, x = tmp[1])))
x.vars <- names(unlist(sapply(colnames(CalibrationData),
                              grep, x = tmp[2])))
if(SUBSET != ""){
  calData <- eval(call("subset", x =  CalibrationData,
                       subset = parse(text = SUBSET)))
}else{
  calData <- CalibrationData
}
calData <- calData[,c(y.var, x.vars)]
calData
```

```
##       Mean1.red    Mean2.red IL6
## 1  0.3704499682 0.0005412395   0
## 2  0.0066671932 0.3188788634  20
## 3  0.1087585316 0.2745492033  15
## 4  0.3724102167 0.0218865865   2
## 5  0.2236482682 0.1782995458  10
## 6  0.3139635361 0.0582015909   4
## 7  0.2325920061 0.0938725607   6
## 8  0.2198977397 0.1440751763   8
## 9  0.2649201802 0.1320164714   6
## 10 0.1291955184 0.1012312192   8
```

```
## 11 0.1007636009 0.1951674212  10
## 12 0.2642769326 0.0863933768   4
## 13 0.0623311351 0.2674246557  15
## 14 0.3304102055 0.0510682096   2
## 15 0.3120236323 0.0068564011   0
## 16 0.0003743322 0.3942393906  20
```

2 Model

We will apply the following model.

```
FORMULA
```

```
## [1] "(Mean2.red / (Mean1.red + Mean2.red)) ~ IL6"
```

3 Analysis

We now fit (simple) linear model.

```
fit <- lm(as.formula(FORMULA), data = calData)
summary(fit)
```

```
##
## Call:
## lm(formula = as.formula(FORMULA), data = calData)
##
## Residuals:
##      Min       1Q   Median       3Q      Max
## -0.06746 -0.03410 -0.01079  0.02363  0.14846
##
## Coefficients:
##             Estimate Std. Error t value Pr(>|t|)
## (Intercept) 0.011896   0.022443    0.53    0.604
## IL6         0.049915   0.002184   22.86 1.75e-12 ***
## ---
## Signif. codes:  0 '***' 0.001 '**' 0.01 '*' 0.05 '.' 0.1 ' ' 1
##
## Residual standard error: 0.05497 on 14 degrees of freedom
## Multiple R-squared:  0.9739, Adjusted R-squared:  0.972
## F-statistic: 522.5 on 1 and 14 DF,  p-value: 1.747e-12
```

We determine the inverse of the fitted model.

```
ab <- coef(fit)
names(ab) <- NULL
predFunc <- function(newdata){}
body(predFunc) <- substitute({ with(newdata, (eval(y)-a)/b) },
                             list(y = parse(text = respVar),
                                a = ab[1],
                                b = ab[2]))
```

We plot the given concentrations against the fitted values.

```
library(ggplot2)
modelPlot <- ggplot(calData, aes_string(x = concVar, y = respVar)) +
            geom_point() + geom_smooth(method = "lm") +
```

```
           annotate("text",  x=-Inf, y = Inf,
           label = substitute(paste(R^2, " = ", R2, ", adj. ", R^2, " = ", adj.R2),
                             list(R2 = signif(summary(fit)$r.squared,3),
                                  adj.R2 = signif(summary(fit)$adj.r.squared, 3))),
           vjust=1, hjust=0, size = 5)
modelPlot
```

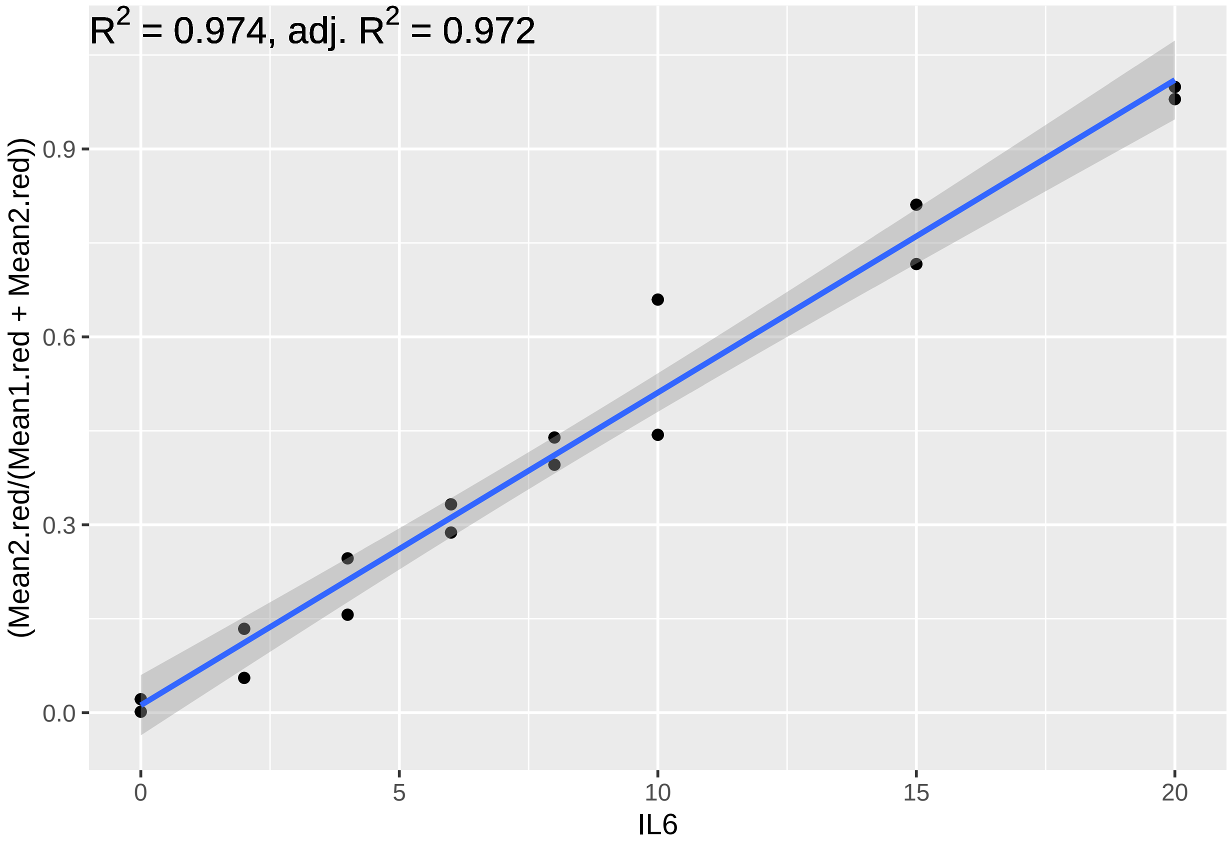

4 Computation of LOB, LOD and LOQ

We compute limit of blank (LOB), limit of detection (LOD) and limit of quantification (LOQ) by inverting the regression fit. We get the LOB by inverting the upper bound of the one-sided 95% confidence interval at concentration 0. In case of LOD, the upper-bound of the 99.95% confidence interval at concentration 0 is inverted. LOQ is determined as $3 \times$ LOD.

```
if(ab[2] > 0){
  LOB <- (confint(fit, parm = 1, level = 0.90)[2]-ab[1])/ab[2]
  LOD <- (confint(fit, parm = 1, level = 0.999)[2]-ab[1])/ab[2]
}else{
  LOB <- (confint(fit, parm = 1, level = 0.90)[1]-ab[1])/ab[2]
  LOD <- (confint(fit, parm = 1, level = 0.999)[1]-ab[1])/ab[2]
}
names(LOB) <- "LOB"
names(LOD) <- "LOD"
LOQ <- 3*LOD
names(LOQ) <- "LOQ"
LOB
```

```
##       LOB
## 0.7919341
```

```
LOD
```

```
##      LOD
## 1.861664
```

```
LOQ
```

```
##      LOQ
## 5.584991
```

5 Save Results

We save the results.

```
save(fit, LOB, LOD, LOQ, file = file.path(PATH.OUT, paste0(FILENAME, "_Results.RData")))
```

6 Save Model

We save the inverse of the fitted model to be able to apply it for predicting concentrations.

```
saveRDS(object = predFunc, file = file.path(PATH.OUT, paste0(FILENAME, "_Model.rds")))
```

7 Software

```
sessionInfo()
```

```
## R version 4.1.2 (2021-11-01)
## Platform: x86_64-pc-linux-gnu (64-bit)
## Running under: Arch Linux
##
## Matrix products: default
## BLAS:   /usr/lib/libopenblasp-r0.3.19.so
## LAPACK: /usr/lib/liblapack.so.3.10.0
##
## locale:
##  [1] LC_CTYPE=en_US.UTF-8       LC_NUMERIC=C
##  [3] LC_TIME=en_US.UTF-8        LC_COLLATE=en_US.UTF-8
##  [5] LC_MONETARY=en_US.UTF-8    LC_MESSAGES=en_US.UTF-8
##  [7] LC_PAPER=en_US.UTF-8       LC_NAME=C
##  [9] LC_ADDRESS=C               LC_TELEPHONE=C
## [11] LC_MEASUREMENT=en_US.UTF-8 LC_IDENTIFICATION=C
##
## attached base packages:
## [1] stats     graphics  grDevices utils     datasets  methods   base
##
## other attached packages:
## [1] ggplot2_3.3.5 shiny_1.7.1
##
## loaded via a namespace (and not attached):
##  [1] Rcpp_1.0.8          locfit_1.5-9.4      lattice_0.20-45
##  [4] fftwtools_0.9-11    png_0.1-7           assertthat_0.2.1
```

```
##  [7] digest_0.6.29       utf8_1.2.2          mime_0.12
## [10] R6_2.5.1            tiff_0.1-10         evaluate_0.14
## [13] highr_0.9           pillar_1.7.0        rlang_1.0.1
## [16] fontawesome_0.2.2   jquerylib_0.1.4     Matrix_1.3-4
## [19] DT_0.20             rmarkdown_2.11      labeling_0.4.2
## [22] shinythemes_1.2.0   splines_4.1.2       shinyjs_2.1.0
## [25] LFApp_1.3           stringr_1.4.0       htmlwidgets_1.5.4
## [28] tinytex_0.35        RCurl_1.98-1.5      munsell_0.5.0
## [31] xfun_0.29           compiler_4.1.2      httpuv_1.6.5
## [34] pkgconfig_2.0.3     BiocGenerics_0.40.0 mgcv_1.8-38
## [37] htmltools_0.5.2     tidyselect_1.1.1    tibble_3.1.6
## [40] fansi_1.0.2         withr_2.4.3         crayon_1.4.2
## [43] dplyr_1.0.7         later_1.3.0         bitops_1.0-7
## [46] grid_4.1.2          nlme_3.1-153        jsonlite_1.7.3
## [49] xtable_1.8-4        gtable_0.3.0        lifecycle_1.0.1
## [52] DBI_1.1.2           magrittr_2.0.2      scales_1.1.1
## [55] stringi_1.7.6       cli_3.1.1           cachem_1.0.6
## [58] shinyMobile_0.9.1   farver_2.1.0        fs_1.5.2
## [61] promises_1.2.0.1    bslib_0.3.1         ellipsis_0.3.2
## [64] shinyFiles_0.9.1    generics_0.1.1      vctrs_0.3.8
## [67] EBImage_4.36.0      tools_4.1.2         glue_1.6.1
## [70] purrr_0.3.4         crosstalk_1.2.0     jpeg_0.1-9
## [73] abind_1.4-5         fastmap_1.1.0       yaml_2.2.2
## [76] colorspace_2.0-2    knitr_1.36          sass_0.4.0
```
